# *Arabidopsis* HDA6 Regulates Locus-Directed Heterochromatin Silencing in Cooperation with MET1

**DOI:** 10.1371/journal.pgen.1002055

**Published:** 2011-04-28

**Authors:** Taiko Kim To, Jong-Myong Kim, Akihiro Matsui, Yukio Kurihara, Taeko Morosawa, Junko Ishida, Maho Tanaka, Takaho Endo, Tetsuji Kakutani, Tetsuro Toyoda, Hiroshi Kimura, Shigeyuki Yokoyama, Kazuo Shinozaki, Motoaki Seki

**Affiliations:** 1 Plant Genomic Network Research Team, RIKEN Plant Science Center, Yokohama, Kanagawa, Japan; 2 Graduate School of Science, The University of Tokyo, Tokyo, Japan; 3 Bioinformatics and Systems Engineering Division, RIKEN Yokohama Institute, Yokohama, Kanagawa, Japan; 4 Department of Integrated Genetics, National Institute of Genetics, Mishima, Shizuoka, Japan; 5 Graduate School of Frontier Biosciences, Osaka University, Suita, Osaka, Japan; 6 Gene Discovery Research Group, RIKEN Plant Science Center, Yokohama, Kanagawa, Japan; 7 Kihara Institute for Biological Research, Yokohama City University, Yokohama, Kanagawa, Japan; University of Cambridge, United Kingdom

## Abstract

Heterochromatin silencing is pivotal for genome stability in eukaryotes. In *Arabidopsis*, a plant-specific mechanism called RNA–directed DNA methylation (RdDM) is involved in heterochromatin silencing. Histone deacetylase HDA6 has been identified as a component of such machineries; however, its endogenous targets and the silencing mechanisms have not been analyzed globally. In this study, we investigated the silencing mechanism mediated by HDA6. Genome-wide transcript profiling revealed that the loci silenced by HDA6 carried sequences corresponding to the RDR2-dependent 24-nt siRNAs, however their transcript levels were mostly unaffected in the *rdr2* mutant. Strikingly, we observed significant overlap of genes silenced by HDA6 to those by the CG DNA methyltransferase MET1. Furthermore, regardless of dependence on RdDM pathway, HDA6 deficiency resulted in loss of heterochromatic epigenetic marks and aberrant enrichment for euchromatic marks at HDA6 direct targets, along with ectopic expression of these loci. Acetylation levels increased significantly in the *hda6* mutant at all of the lysine residues in the H3 and H4 N-tails, except H4K16. Interestingly, we observed two different CG methylation statuses in the *hda6* mutant. CG methylation was sustained in the *hda6* mutant at some HDA6 target loci that were surrounded by flanking DNA–methylated regions. In contrast, complete loss of CG methylation occurred in the *hda6* mutant at the HDA6 target loci that were isolated from flanking DNA methylation. Regardless of CG methylation status, CHG and CHH methylation were lost and transcriptional derepression occurred in the *hda6* mutant. Furthermore, we show that HDA6 binds only to its target loci, not the flanking methylated DNA, indicating the profound target specificity of HDA6. We propose that HDA6 regulates locus-directed heterochromatin silencing in cooperation with MET1, possibly recruiting MET1 to specific loci, thus forming the foundation of silent chromatin structure for subsequent non-CG methylation.

## Author Summary

Eukaryotes are defended from potentially harmful DNA elements, such as transposons, by forming inactive genomic structure. Chromatin, which consists of DNA and histone proteins, is densely packed in the silent structure, and chromatin chemical modifications such as DNA methylation and histone modifications are known to be essential for this packing. In plants, small RNA molecules have been thought to trigger DNA methylation and resulting silent chromatin formation. We revealed that elimination of specific histone modifications concomitant with DNA methylation is pivotal for the silent chromatin. Furthermore, the histone deacetylase was shown to have more profound target specificity than the DNA methyltransferase and is required for locus-directed DNA methylation, implying the involvement of the histone deacetylase for targeting the DNA methyltransferase to specific places on the genome. These proteins and their functions for gene silencing are evolutionarily conserved in higher eukaryotes, and several proteins involved in small RNA production are plant-specific. Thus, we present a hypothesis that the plant genome may build the protecting foundation by the conserved genome surveillance in eukaryotes, and the reinforcing machinery involving small RNAs could be evolutionarily added to the plant heterochromatin silencing system.

## Introduction

Chromatin modification is epigenetic information that has evolved in diverse eukaryotes adding another layer of information to the DNA code. In higher eukaryotes, histone modification and DNA methylation are involved in numerous biological processes such as development, regeneration, and oncogenesis [1], [2]. In addition, the eukaryotic genome has evolved epigenetic mechanisms to silence potentially harmful transposable elements (TEs) and the repetitive elements that constitute a large proportion of the genome [3]. Heterochromatin formation, a striking function of the eukaryotic genome, is intricately controlled through repressive histone modification and DNA methylation [4]. Thus, mutations that affect the status of chromatin structure often result in strong phenotypic alterations or inviability, because of aberrant regulation of gene expression or distorted genome stability [5]–[8].

The flowering plant, *Arabidopsis thaliana*, is a model organism particularly suited for epigenetic research due to the availability of viable and heritable null mutants of histone modifying enzymes and DNA methyltransferases. Recent genome-wide studies on epigenetic marks of gene silencing in plants have focused on DNA methylation or repressive histone methylations [9]–[13]. Few studies, however, have focused on histone deacetylation, which is crucial for epigenetic regulation in eukaryotes [14], [15]. Investigating histone deacetylation and DNA methylation in *Arabidopsis* could contribute not only to our understanding of plant biology, but also to a broad range of essential biological processes in mammals and therapeutic applications in humans [16], [17].

Gene silencing has been investigated extensively in *Arabidopsis*. Plants have evolved gene silencing machinery called RNA-directed DNA methylation (RdDM). Plant-specific RNA POLYMERASE IV (Pol IV), RNA-DEPENDENT RNA POLYMERASE 2 (RDR2) and DICER-LIKE 3 (DCL3) are involved in the production of 24-nt small interfering RNAs (siRNAs) that guide DNA methyltransferases, DOMAINS REARRANGED METHYLTRANSFERASES 1/2 (DRM1/2), to the corresponding genomic DNA for *de novo* DNA methylation in all cytosine contexts (CG, CHG, CHH; H: A, T, or C; [18]). METHYLTRANSFERASE 1 (MET1), a homolog of mammalian DNMT1, is primarily responsible for the maintenance of genome-wide CG methylation [19]–[22]. KRYPTONITE (KYP), a member of the Su(var)3–9 class of histone methyltransferases, contributes an epigenetic mark of constitutive heterochromatin, histone H3 Lys 9 dimethylation (H3K9me2) [23], [24]. CHROMOMETHYLASE 3 (CMT3), a plant-specific DNA methyltransferase, maintains CHG methylation via H3K9me2 dependence mediated by KYP [23], [25], [26]. Histone Deacetylase 6 (HDA6), a homolog of yeast RPD3 and mammalian HDAC1, is involved in gene silencing and RNA-directed DNA methylation [27]–[30].

Of the 16 *Arabidopsis* histone deacetylases [31], the importance of HDA6 in gene silencing was discovered by identification of *HDA6* in three independent genetic screens of gene silencing [27], [32], [33]. In each case, *hda6* mutant plants lacking histone deacetylase activity (*sil1*, *axe1*, and *rts1*) were shown to exhibit reactivation of transcription on target transgenes. Analyses of the endogenous function of HDA6 have been limited, thus far, to the regulation of chromatin at repetitive sequences such as rDNA loci [28], [30], [34], [35], transposable elements and centromeric satellite repeats [29], [36]. However, the positions of the loci silenced by HDA6 have yet to be determined genome-wide.

Various effects of the *hda6* mutations on cytosine methylation have been observed previously. Several transposable elements were hypomethylated in *sil1* [36]. Reduction of DNA methylation has been reported for the siRNA-directed NOS promoter in *rts1*, predominantly at CG and CHG sites [27]. Similarly, a reduction in CG and CHG methylation was observed in *axe1-5*, *sil1*, and *rts1* mutants at rDNA repeats, although the demethylation was much less than that observed in the DNA hypomethylation mutant *ddm1* [28], [30]. In contrast to these observations, a drastic reduction in CHG methylation, but not CG methylation, was observed in a Sadhu-type transposable element in *axe1-5* [37], and 5S rDNA in *sil1* [35]. Furthermore, few changes in DNA methylation were observed in the centromeric repeats or transgene region in *sil1*, although their silencing was lost [29], [38]. These various effects of the *hda6* mutations on DNA methylation might be due to locus dependence rather than differences in the mutations themselves, because similar effects were observed between the mutants [28], [30]. Because previous studies have focused on only a few specific loci, precisely how the *hda6* mutation influences DNA methylation in general remains obscure. Therefore, a genome-wide analysis of HDA6 target loci is vital to improve our understanding of the mechanistic basis for HDA6-mediated gene silencing via DNA methylation and histone modification.

In this study, aimed at understanding the silencing mechanism mediated by HDA6, we identified HDA6 transcriptionally repressed loci across the genome and determined the direct targets of HDA6. We also studied the regulation mechanisms involved in histone modification and DNA methylation on HDA6 direct targets. Our data show that the *hda6* mutation causes loss of heterochromatic marks and aberrant enrichment for euchromatic epigenetic marks at HDA6 direct targets. Furthermore, we present evidence that the upregulated loci in *hda6* overlapped with those in *met1*, and that the *hda6* mutation causes the complete loss of DNA methylation on some HDA6 target loci. These results suggest that a strong functional connection between HDA6 and MET1 exists. Remarkably, hypomethylation only occurred in *hda6* on the HDA6 target loci where surrounding MET1 targets were absent. We propose, therefore, that HDA6 is required for gene silencing and that it acts in cooperation with MET1 to build the infrastructure of heterochromatin.

## Results

### Genome-Wide Identification of Loci Derepressed in *axe1-5*

**Figure 1:**
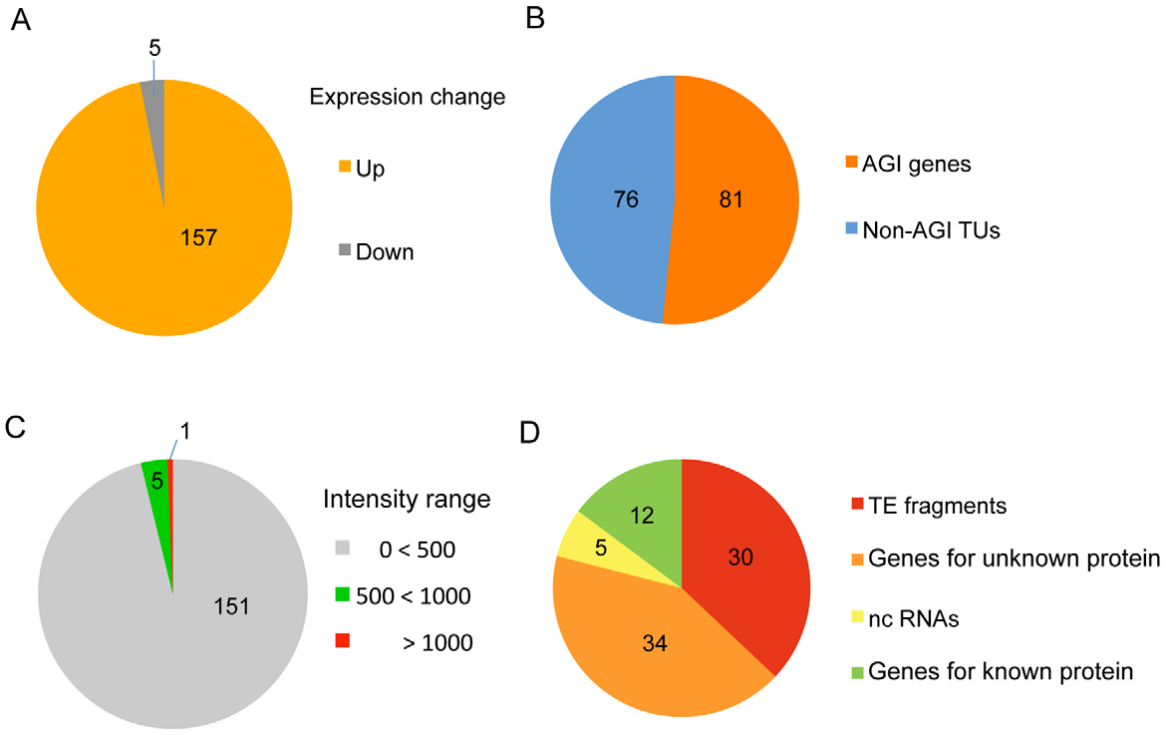
HDA6 is required for genome-wide gene silencing of TE fragments and other silenced loci. Transcript profiling of the *hda6* mutant, *axe1-5*, using a tiling array revealed that the *hda6* mutation resulted in derepression of 157 loci including TE fragments and other silenced regions. (A) The fractions of the loci upregulated (orange) or downregulated (grey) in *axe1-5*. The 157 upregulated loci accounted for 97% of differentially expressed loci. (B) The fraction of AGI genes (red) and non-AGI TUs (blue) out of the loci upregulated in *axe1-5*. (C) The fraction of silenced loci (signal intensity <500) in wild-type plants (grey) out of the loci upregulated in *axe1-5*. (D) Functional classification of upregulated AGI genes in *axe1-5*. TE fragments (red; 37%); genes for unknown proteins (orange; 42%); ncRNA (yellow; 6%); genes for known proteins (green; 15%).

To identify target loci for HDA6 binding in the *Arabidopsis* genome, we first performed a genome-wide comparison of RNA accumulation between the wild-type plant (DR5) and the *hda6* mutant, *axe1-5* [33], using a whole-genome tiling array. This approach identified 157 statistically significant loci that were transcriptionally upregulated in *axe1-5* compared with wild-type plants (>3 fold, p-initial<10^−6^, FDR α = 0.05) (Figure 1A and 1B). RT–PCR of a random selection of these loci was used to confirm their up-regulation in the *axe1-5* mutant (Figure S1). Among these loci, nearly half (81 genes; see Table S1) were annotated by the Arabidopsis Genome Initiative (http://www.arabidopsis.org/; hereafter referred to as AGI genes). The other half (76 genes; Table S2) were intergenic non-AGI annotated transcriptional units (non-AGI TUs) identified using the ARTADE program [39] (Figure 1B). It is noteworthy that only a small fraction of transcripts (5 loci: 3% of all differentially expressed loci) were classified as having reduced levels of expression in *axe1-5* (Figure 1A, Table S3). We consistently found that the loci upregulated in *axe1-5* were strongly silenced in wild-type plants (Figure 1C) and consisted predominantly of TE fragments and genes for unknown proteins (79%) (Figure 1D). A survey of the TE fragments [40], mapping on or around the loci upregulated in *axe1-5* (from 1 kb upstream to 1 kb downstream), showed that a significant number of the fragments (342 TE fragments) were located on or around such loci (Table S1 and S2). These results show that HDA6 regulates gene silencing on a genome-wide scale.

### HDA6-Mediated Gene Silencing Is Mostly Independent of the RdDM Components

**Figure 2:**
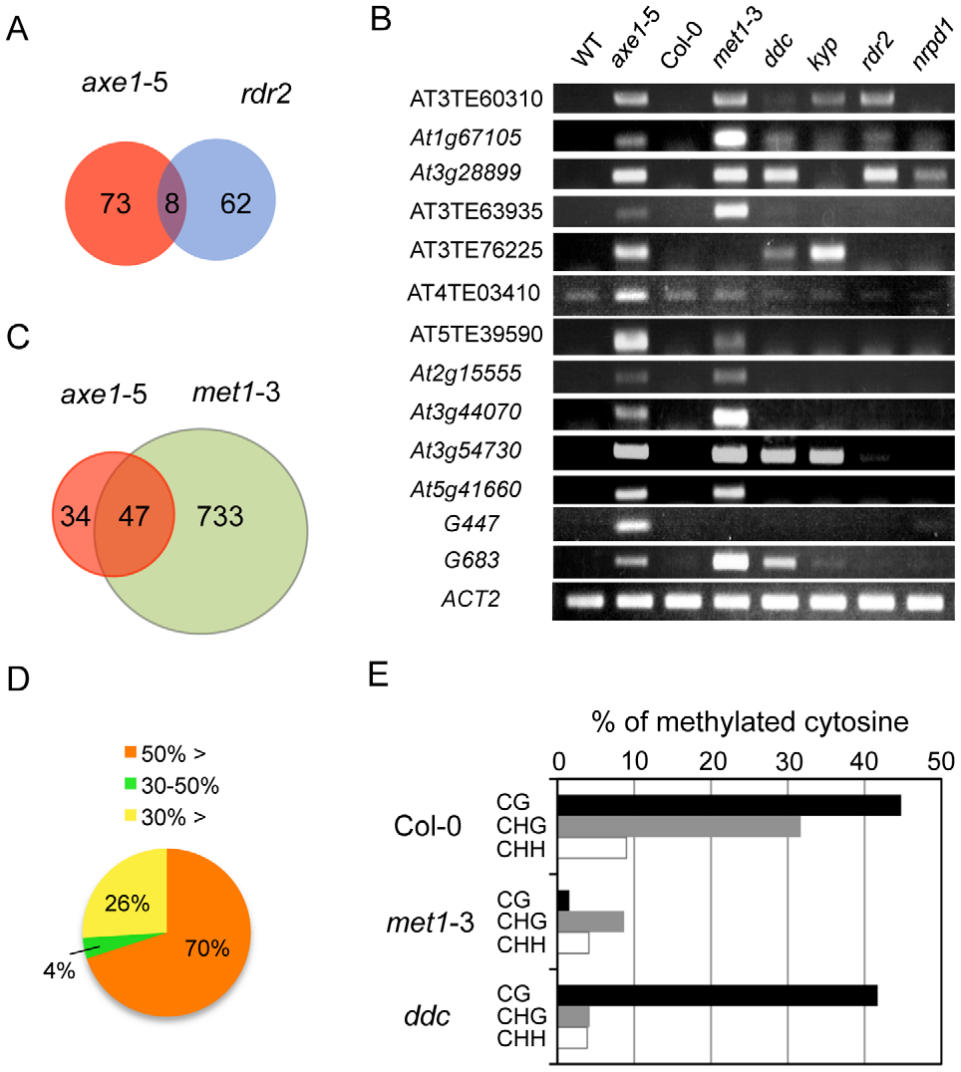
HDA6-mediated gene silencing requires MET1 but not RdDM pathway. (A) A Venn diagram showing the small fraction of overlap between the AGI genes upregulated in *axe1-5*, compared with those in *rdr2*. The AGI genes identified as upregulated in *rdr2* in a previous genome-wide transcriptional profiling experiment [43], were compared with the AGI genes upregulated in *axe1-5* identified in this study. (B) Various mutants deficient in gene silencing, such as the chromatin modifying enzymes (*hda6*, *met1*, *ddc*, and *kyp*) and siRNA production (*rdr2* and *nrpd1*) were examined for the activation of representative HDA6 target loci (AT3TE60310, *At1g67105*, *At3g28899*, AT3TE63935, AT3TE76225, AT4TE03410, AT5TE39590, *At2g15555*, *At3g44070*, *At3g54730*, *At5g41660*, *G447* and *G683*) by RT-PCR analysis. *ACT2* served as a control. The MET1 requirement for repression of *HDA6* target loci is highlighted. (C) A Venn diagram showing the significant overlap between the AGI genes upregulated in *axe1-5* and in *met1-3* that were identified in genome-wide transcriptional profiling. (D) The DNA methylation status in wild-type Col-0 for the genes upregulated in *axe1-5*. The fraction of AGI genes associated with DNA methylation at more than 50% of methylated cytosines around TSS (−500 to +500 from TSS) was determined using publicly available datasets of methylcytosine immunoprecipitation [13]. (E) Cytosine methylation was investigated in each sequence context (CG, CHG, and CHH) in wild-type Col-0, *met1-3*, and *ddc* using publicly available datasets of cytosine methylation (methylC-seq, [13]). The percentages of methylated cytosines of the AGI genes upregulated in *axe1-5* are shown.

Forward genetic screens for plants deficient in RNA-mediated transcriptional silencing identified *HDA6* as an essential component of the RdDM pathway [27], [41]. To address whether the endogenous HDA6 target loci were also directed by the RdDM pathway, siRNAs from the ASRP database [42] were mapped to the loci derepressed in *axe1-5*. Consistent with knowledge that 24-nt-long siRNAs are required for the establishment of RdDM, the most abundant siRNAs mapping to upregulated loci in *axe1-5* are 24-nt long (Figure S2A). These 24-nt siRNAs were hardly found in the *rdr2* and *dcl3* mutants (Figure S2B), suggesting that loci derepressed in *axe1-5* contain siRNA sequences produced by RDR2 and DCL3-dependent pathways, as previously predicted [27], [41]. Thus, our previous study for the targets of RDR2 [43], that were identified using same growth conditions and array technology as in this study, were compared with the genes derepressed in *axe1-5*. This revealed, surprisingly, despite the loss of 24-nt siRNAs from those genes were observed in the *rdr2* mutant (Figure S2B), the majority of the loci derepressed in *axe1-5* are kept in a silenced state in the *rdr2* mutant (Figure 2A). In fact, elevated transcript levels were not detectable at many loci in the mutants deficient in siRNA production (*rdr2*, and *nrpd1*; 10 and 11 respectively, out of 13 genes tested; Figure 2B). There also was evidence that small subsets of the HDA6-mediated gene silencing showed dependence on RdDM pathway and the overlapped genes between *axe1-5* and *rdr2* was confirmed for their accumulated transcripts (AT3TE60310, *At1g67105*, and *At3g28899*) in the RdDM mutants (*rdr2* and *nrpd1*; Figure 2B). Interestingly, larger overlap to the triple mutant *drm1 drm2 cmt3* (*ddc*) involved in siRNA-directed non-CG methylation was observed (5 out of 13 genes; Figure 2B). Taken together, these results indicate the partial involvement of RdDM pathway in HDA6-mediated endogenous gene silencing.

### HDA6 and the CG DNA Methyltransferase MET1 Share Common Target Loci for Epigenetic Silencing

We also examined the effects of mutations in other chromatin modifying enzymes on the silencing of putative HDA6 target loci. Strikingly, 10 out of 13 of the putative HDA6 target loci were also upregulated in the *met1-3* mutant (Figure 2B). To address whether HDA6 and MET1 share common target loci genome-wide, we also identified differentially regulated loci in *met1-3* using a tiling array (Tables S4, S5, S6 and S7), and compared the upregulated loci in *met1-3* with those in *axe1-5*. A significant overlap of upregulated loci in *axe1-5* to those in *met1-3* was observed (Hypergeometric distribution, P = 1.08E^−54^; Figure 2C). Furthermore, the DNA methylation status of the loci derepressed in *axe1-5* was also investigated using publicly available DNA methylation datasets [13]. Most of the genes upregulated in *axe1-5* (i.e. 70% of the upregulated AGI genes) were substantially methylated in the wild-type plants with more than 50% of all cytosines at regions surrounding transcriptional start sites methylated (Figure 2D). Cytosine methylation in the wild-type plants was predominantly found at CG, to a lesser extent at CHG, and least of all at the CHH sites of derepressed AGI genes in *axe1-5* (Figure 2E). A large proportion of the cytosine methylation on derepressed AGI genes in *axe1-5* appears to be highly dependent on MET1 because the drastic reduction in cytosine methylation was observed not only at CG, but also CHG and CHH sites (Figure 2E). In contrast, CG methylation in the *ddc* mutant remained at similar levels as the wild-type plants (Figure 2E). Thus, these data demonstrate that the CG DNA methyltransferase MET1 is required for HDA6-mediated epigenetic gene silencing.

### Identification of the Direct Targets of HDA6

**Figure 3:**
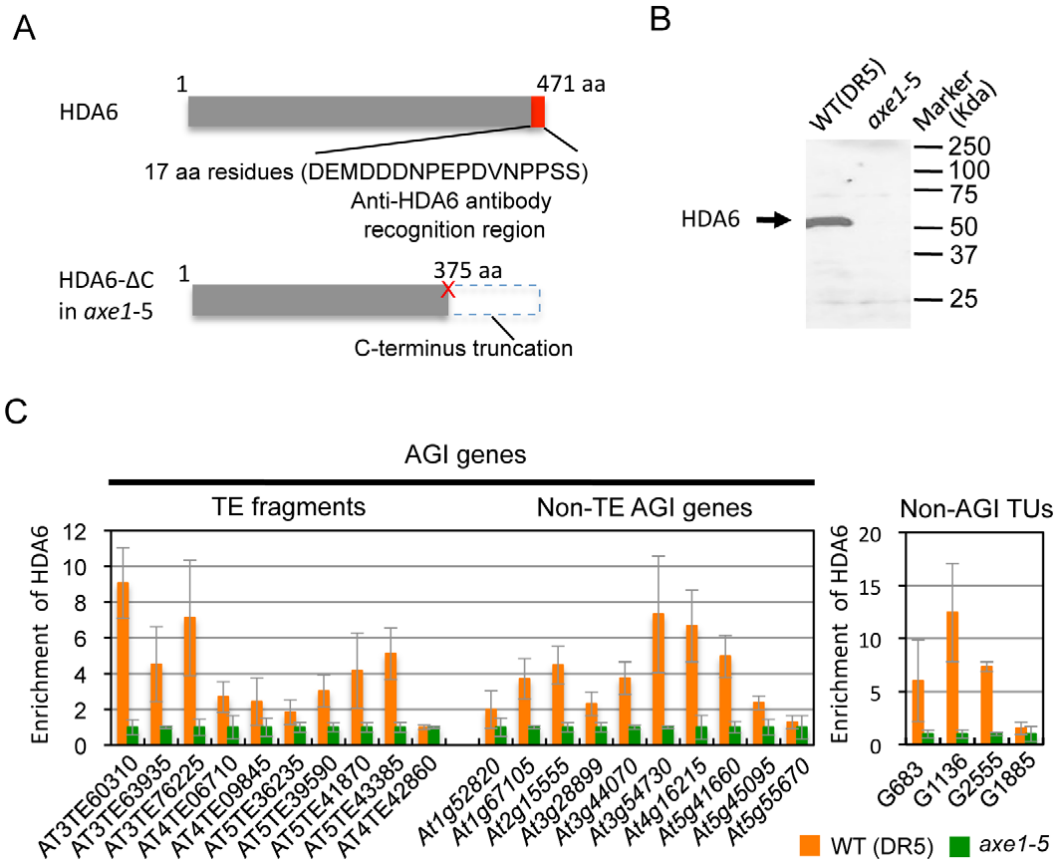
Identification of direct targets of HDA6 using an HDA6-specific antibody. (A) Schematic illustration of HDA6-specific antibody raised in this study. The point mutation in *axe1-5* [33], is indicated by a red cross and the resulting C-terminal truncation of the HDA6 protein in *axe1-5* is indicated by a dashed box. The 17 amino acid (aa) peptide selected for the epitope is indicated by a red box, which is absent in *axe1-5*. (B) Western blot analysis indicates high specificity of the HDA6 antibody for its target. Total protein extracts from wild-type (WT) and *axe1-5* plants were analyzed to check the specificity of the HDA6 antibody. (C) Screening of HDA6 direct targets by ChIP-qPCR assay. The transcribed regions of 22 selected loci from those derepressed in *axe1-5* (Figure S1), including 9 AGI annotated TE fragments, 9 non-TE AGI genes and 4 non-AGI TUs were used in this study. AGI annotated TE fragments are as follows: AT3TE60310 (*At3g42658*), AT3TE63935 (*At3g44042*), AT3TE76225 (*At3g50625*), AT4TE06710 (*At4g02960*), AT4TE09845 (*At4g04293*), AT5TE36235 (*At5g27927*), AT5TE39590 (*At5g28880*), AT5TE41870 (*At5g30480*), and AT5TE43385 (*At5g32566*). The loci down-regulated in *axe1-5* (AT4TE42860: *At4g16870*), and a gene with no apparent transcriptional difference between *axe1-5* and WT (*At5g55670*) were included in this screening. Equal amounts of the input DNA and the immunoprecipitates were analyzed and normalized against the input DNA. The values obtained were normalized with *ACT2* and the relative enrichment of HDA6-binding in the wild-type plants compared with *axe1-5* is shown as the mean and standard deviations obtained from three independent immunoprecipitates (orange, WT; green, *axe1-5*).

Identification of the direct targets of HDA6 is a crucial step in providing mechanistic insight into HDA6 function in transcriptional control, chromatin regulation, and DNA methylation. To determine the HDA6 target loci using chromatin immunoprecipitation (ChIP), we raised a specific antibody against HDA6. The epitope for the antibody was designed against the C-terminal region of the HDA6 protein that is absent in *axe1-5*. We verified that the peptide sequence was not similar to any other sequence of annotated *Arabidopsis* proteins. Thus, comparisons made between wild-type and *axe1-5* for the level of enrichment of immunoprecipitated DNA using the antibody allowed us to exclude any non-specific signals to identify HDA6 binding sites (Figure 3A). Western blot analysis confirmed the specificity of the antibody, which detected a unique band of 53 kDa in the wild-type, but not in *axe1-5* (Figure 3B).

Using the HDA6 antibody, we performed ChIP assays and quantitative PCR (qPCR). Three genes, AT3TE60310 (*At3g42658*), AT3TE76225 (*At3g50625*) and *At5g41660* were selected from genes upregulated in *axe1-5*, representing RdDM dependent, MET1 independent, and MET1 dependent genes, respectively (Figure S1). Three primer sets were designed for each gene within the promoter, 5′ and 3′ regions of the genes (Figure S3). HDA6 binding levels in the wild-type plants were significantly higher than in *axe1-5* for all of the genes tested, regardless of the dependence on RdDM pathway or MET1. This indicates that HDA6 binds directly to all such genes. In addition, preferential binding of HDA6 was observed within the 5′ regions of the genes. Therefore, a further screening of HDA6 target loci was performed for the 5′ regions of selected loci from those upregulated in *axe1-5* (Figure S1; Figure 3C). As a negative control, we tested one gene that exhibited no apparent transcriptional change (*At5g55670*, Figure S1), and found only a minimal difference. Our experiments show that HDA6 binding levels were enriched by 2 to 12-fold in the wild-type plants relative to *axe1-5*, with statistical significance observed for 17 of the loci (Figure 3C). 5 loci did not show significant differences between the wild-type and *axe1-5* mutant.

### HDA6 Is Required for Heterochromatic Silencing, and the Mutation Results in Loss of Heterochromatic Histone Modification Along with Aberrant Enrichment for Euchromatic Modification at HDA6 Targets

**Figure 4:**
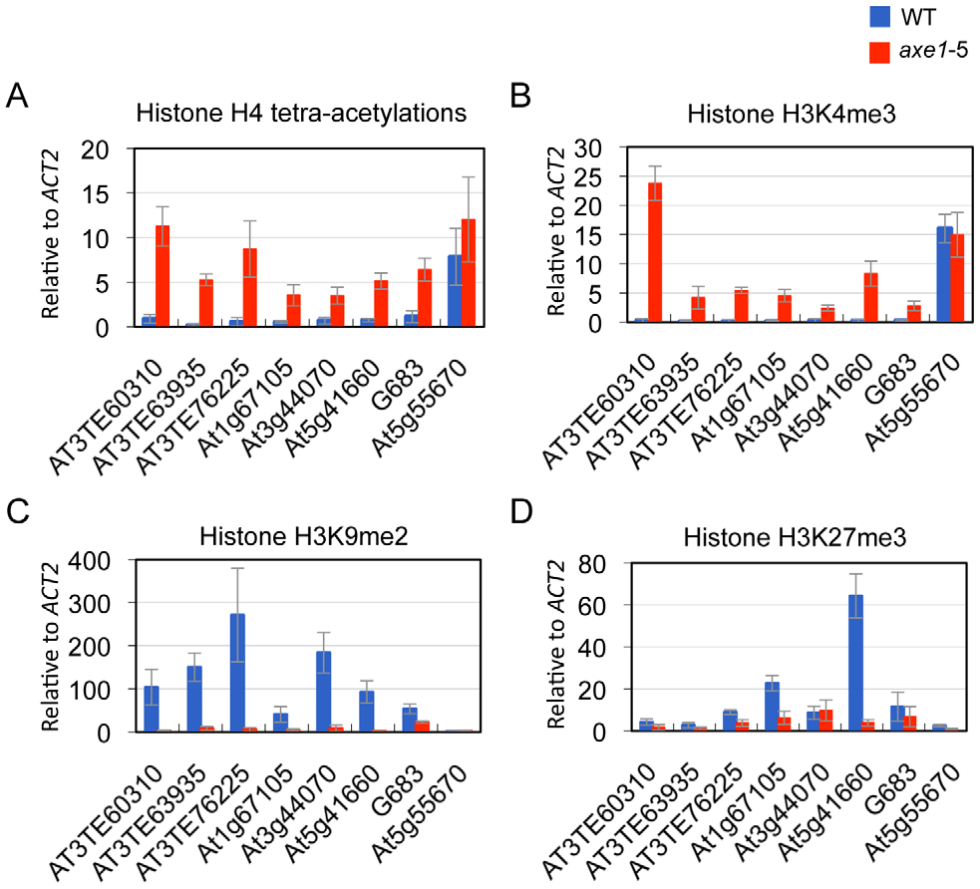
*HDA6* mutation causes abolishment of heterochromatic mark and elevation of euchromatic modifications. ChIP-qPCR assays were performed for quantitative analysis of histone modifications, using antibodies against; (A) H4 tetra-acetylation, (B) H3K4me3, an active euchromatic mark, (C) H3K9me2, and (D) H3K27me3, a constitutive heterochromatic or repressive mark. Equal amounts of input DNA and the immunoprecipitates were analyzed and normalized against input DNA. The values obtained were normalized again using *ACT2* and are shown as the means and standard deviations from three independent immunoprecipitated DNA analyses (blue, WT; red, *axe1-5*). *At5g55670* with no apparent transcriptional difference between *axe1-5* and WT was used as a negative control.

Heterochromatic or repressive regions are associated with H3K9me2 and/or H3K27me3, whereas euchromatic or transcriptionally active regions are associated with H3K4me3 and H4 tetra-acetylation (H4 tetra-acetylated on K5, K8, K12, and K16) [44]. Furthermore, many endogenous RdDM targets are known to be associated with euchromatic modification H3K4me3 [45]. To elucidate HDA6 function and chromatin status, the effects of the *hda6* mutation on histone modification was analyzed using ChIP-qPCR on its direct targets. The results show that, regardless of the dependence on siRNAs or positions on the chromosome, weak H4 tetra-acetylation and H3K4me3 and significantly high levels of heterochromatic modification, H3K9me2, were observed in the wild-type plants at all of the loci tested (Figure 4A, 4B and 4C; Figure S4). In the *axe1-5* mutant, the active marks strongly increased (H4 tetra-acetylation at range 5 to 30 fold and H3K4me3 at 8 to 78 fold, respectively), and the levels of H3K9me2 were drastically reduced (range 2 to 53 fold) compared with the wild-type plants (Figure 4A, 4B and 4C; Figure S4). H3K27me3, another repressive modification, are highly enriched in wild-type plants predominantly on genes within the chromosome arm regions, such as *At1g67105* and *At5g41660*, and drastically reduced in *axe1-5* (Figure 4D). Consistent with this observation, the enrichment of H3K27me3 in the euchromatic arm regions was also seen in the previous genome-wide studies of H3K27me3 [11], [12]. These results indicate that the *hda6* mutation caused an alteration of the chromatin status from a heterochromatic to euchromatic state that was concomitant with transcriptional release of HDA6 target loci.

**Figure 5:**
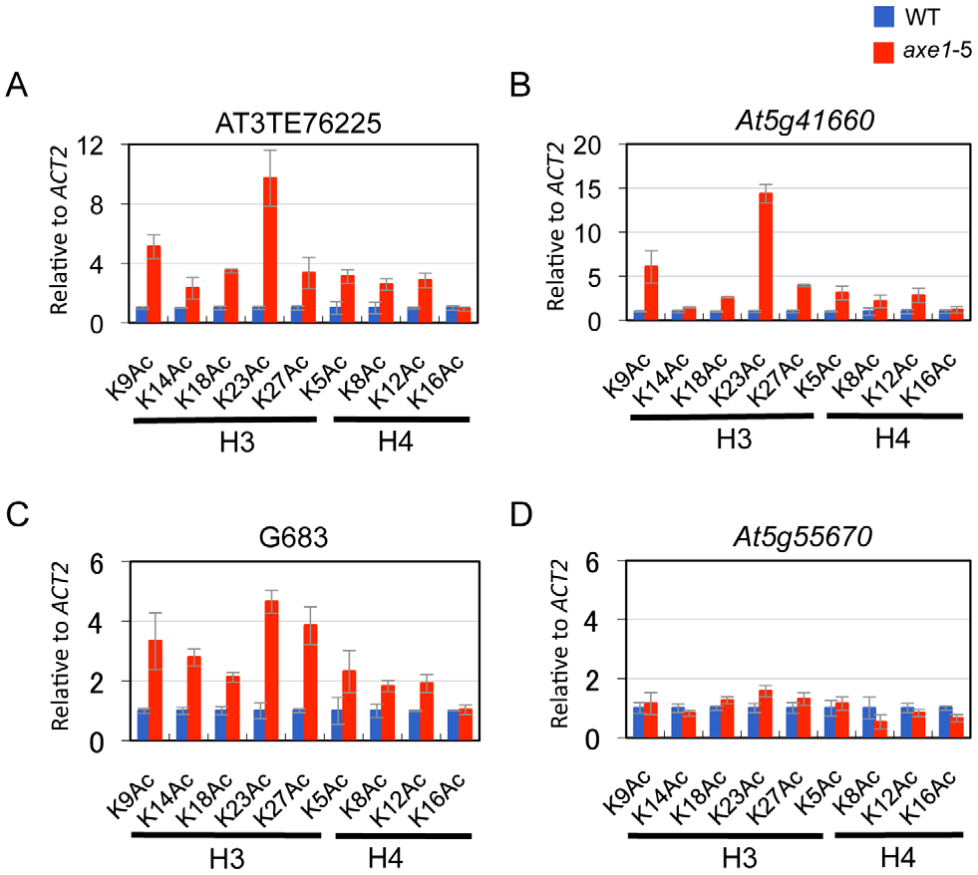
Elevated acetylation at all H3 and H4 lysines except H4K16 in *axe1-5*. ChIP-qPCR assays for all of the potential deacetylation substrates of histone H3 and H4 N-tails (acetylation sites, K9, K14, K18, K23, K27 of H3 and K5, K8, K12, K16 of H4) were examined on (A) AT3TE76225, (B) *At5g41660*, (C) *G683* and a negative control gene (D) *At5g55670*. Specific antibodies against each acetylated lysine were used (See Materials and Methods for detail). The normalized acetylation enrichments in *axe1-5*, relative to those in the wild-type plant are shown as the mean plus standard deviation obtained from three independent immunoprecipitated DNA experiments (blue, WT; red, *axe1-5*).

HDA6 deacetylase activity has been reported for H3K9, H3K14, H4K5 and H4K12, as well as for H4 tetra-acetylation [34]. The activity at other residues, however, is currently unknown. To assess the HDA6 deacetylase activity at other lysine residues, we investigated all of the potential acetylation sites of the H3 and H4 N-tails, including pre-determined sites using ChIP-qPCR. Three HDA6 target loci (AT3TE76225, *At5g41660*, and *G683*) were examined. The results showed that in *axe1-5*, the acetylation levels significantly increased at H3K9, H3K14, H3K18, H3K23, H3K27, H4K5, H4K8, and H4K12 residues relative to those in wild-type plants at all three loci tested (Figure 5A, 5B and 5C, Figure S5). Interestingly, among these residues, H3K23ac levels showed the highest enrichments in *axe1-5*, for the three loci, AT3TE76225, *At5g41660* and *G683* (at 10, 14, 5 fold respectively). The acetylation levels were not significantly altered in *axe1-5* for the control genes *At5g55670* (Figure 5D) and *ACT2* (Figure S5). It is noteworthy that the deacetylase activity observed for HDA6 at H3K27ac as well as H3K9ac, are both likely to be important for the subsequent histone methylation of H3K27me3 and H3K9me2, respectively [46]. Interestingly, residues that showed increased levels of acetylation in *axe1-5*, were identical to the target residues in yeast RPD3 deacetylation [47]. These results indicate that the deacetylase activity of HDA6 occurred on all of the lysine residues in H3 and H4 N-tails, except H4K16.

### The Effect of the *hda6* Mutation on DNA Methylation

**Figure 6:**
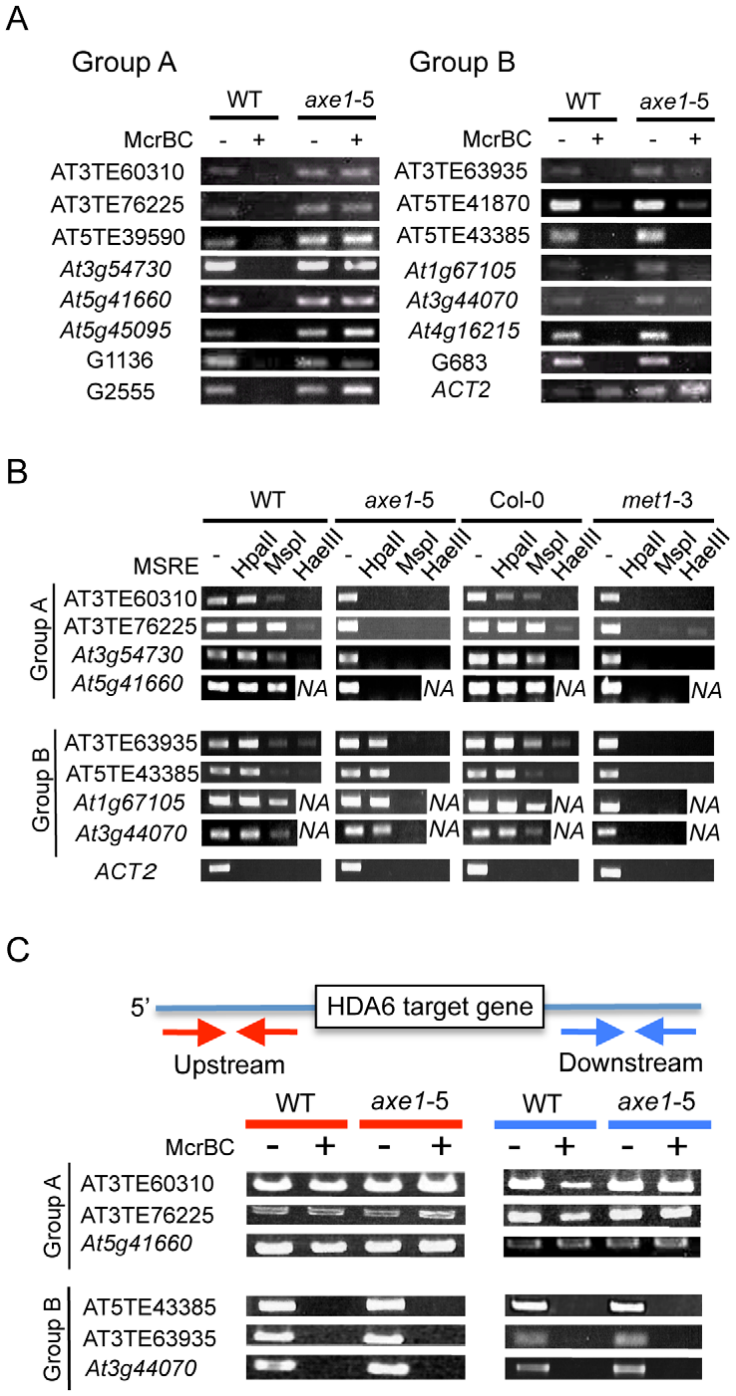
Differential impacts of the *hda6* mutation on DNA methylation at HDA6 target loci. The effect of the *hda6* mutation on cytosine methylation at HDA6 direct targets was determined by; (A) McrBC assays and, (B) Chop-PCR assays. (A) McrBC-digested genomic DNA was amplified by PCR. (B) Chop-PCR assays were conducted using the methylation sensitive restriction enzymes *Hpa*II (which reports CG methylation), *Msp*I (which reports CHG methylation), and *Hae*III (which reports CHH methylation). Genomic DNA digested with each enzyme was amplified by PCR. NA indicates that the amplified sequences do not include the *Hae*III recognition sequence GGCC. In each assay, undigested genomic DNA was used as a PCR control. *ACT2* served as a control for enzymatic digestion. (C) The DNA methylation status for flanking regions of HDA6 target loci was determined using McrBC assays. The regions from around 2 kb to 0.5 kb (i.e. 1.5 kb) of upstream (left panel, red arrows), and downstream (right panel, blue arrows), of HDA6 target loci were assessed. Absence of flanking methylated regions around the HDA6 target loci correlated with the loss of CG methylation in *axe1-5*. Primers are listed in Table S8.

The different effects that *hda6* mutations impose on DNA methylation have been reported as described above. To assess the function of HDA6 on DNA methylation and the relationship between HDA6 and MET1, the DNA methylation status of HDA6 direct targets was investigated. We used the endonuclease McrBC (Figure 6A), which preferentially cleaves methylated DNA, and a Chop-PCR assay using methylation sensitive restriction enzymes (Figure 6B), whose cleavage is blocked by DNA methylation.

In the McrBC assay shown in Figure 6A, no strong bands were detected in the wild-type plants after McrBC digestion, indicating that the direct targets of HDA6 are highly DNA methylated in the wild-type plants. This is consistent with dense DNA methylation on the loci upregulated in *axe1-5* (Figure 2D and 2E). However, in *axe1-5*, we observed two types of McrBC sensitivity among the HDA6 direct target loci. The Group A genes substantially lost cytosine methylation, as demonstrated by the presence of strong bands of similar intensity in both Group A genes and the non-digested control (Figure 6A, left panel). In contrast, the genes in Group B retained DNA methylation in *axe1-5* in common with the wild-type plants as no strong bands were detected (Figure 6A, right panel).

We also performed Chop-PCR assays to investigate in which cytosine contexts are dependent on HDA6 (Figure 6B). The methylation sensitive restriction enzymes used were *Hpa*II, *Msp*I and *Hae*III, which reports CG, CHG, and CHH methylation, respectively. From each group categorized in Figure 6A, four representative HDA6 target loci were tested and using *ACT2* as a negative control. PCR amplification of *ACT2* was undetectable regardless of the genotypes or the enzymes tested (Figure 6B), confirming substantial cleavage by the enzymes had occurred. In the wild-type plants, strong amplification, at a similar level as the undigested control was detected after digestion with *Hpa*II (CG methylation). Strong amplification was observed with *Msp*I (CHG methylation), but it was mostly less than the undigested control; least amplification of all was observed with *Hae*III (CHH methylation) (Figure 6B). The results of this experiment are consistent with the results shown in Figure 2E and Figure 6A, where HDA6 target loci were shown to be significantly methylated in wild-type plants, predominantly at CG sites, and to a similar or lesser extent at CHG sites, but least of all at CHH sites.

In common with the results obtained for the McrBC assay (Figure 6A); the results from the Chop-PCR assays split the HDA6 target loci into two separate groups. In Group A (AT3TE60310, AT3TE76225, *At3g54730* and *At5g41660*), complete demethylation in *axe1-5* occurred in all sequence contexts and amplification after digestion of each enzyme was undetectable (Figure 6B, Group A). In the Group B genes (AT3TE63935, AT5TE43385, *At1g67105*, and *At3g44070*), for *axe1-5*, however, CG methylation was mostly sustained at the similar level as the wild-type (Figure 6B, lower panel, *Hpa*II digest), although a drastic reduction in CHG and CHH methylation was detected [Figure 6B, lower panel CHG (*Msp*I) and CHH (*Hae*III)]. Bisulfite sequencing analyses of wild-type, *axe1-5* and *met1-3* further confirmed that Group A genes (*At5g41660* and AT3TE76225) lost DNA methylation in all sequence contexts in *axe1-5*, and a Group B gene (AT3TE63935) lost CHG and CHH methylation but sustained CG methylation in *axe1-5* (Figure S6). Collectively, the methylation analyses of the HDA6 targets demonstrated that, 1) CG and CHG sites were predominantly highly methylated in the wild-type plants; 2) CHG and CHH methylation was substantially reduced at all target loci in *axe1*-*5*; and, 3) CG methylation was lost at some loci but sustained at others in *axe1-5*.

### An Absence of DNA Methylated Regions around HDA6 Target Loci Is Correlated with the Loss of CG Methylation in *axe1-5*

Why were two different CG methylation states observed in *axe1-5*? Interestingly, according to the public database for DNA methylation [9], a clear correlation was observed between a loss of CG methylation on the HDA6 targets in *axe1-5* and the absence of methylated DNA regions around the HDA6 target loci (Figure S7). HDA6 target loci in Group A (with loss of DNA methylation) were shown to be isolated from other methylated DNA regions, whereas HDA6 target loci in Group B (with persistent CG methylation) were surrounded by other methylated DNA regions. We confirmed the presence or absence of DNA methylated regions around HDA6 target loci (Figure 6C). The 1.5 kb regions upstream and downstream of the HDA6 target loci were analyzed to determine their DNA methylation status using McrBC assays. We found evidence of robust DNA methylation around the HDA6 target loci in Group B (with persistent CG methylation in *axe1-5*) (Figure 6C, Group B). These methylated regions often contained other TE fragments adjacent to HDA6 target loci. These TEs were densely DNA methylated dependently on MET1 but independently of HDA6, since the PCR amplification after McrBC digestion was detected only in *met1-3* (Figure S8). We also confirmed that these adjacent TE fragments were not targeted by HDA6 using ChIP-qPCR assays; these results showed no enrichment of HDA6 binding to adjacent TE fragments in the wild-type plants compared with *axe1-5* (Figure S9). As a result, HDA6 target loci with sustained CG methylation in *axe1-5* (Group B) must harbor the flanking TE fragments that are highly DNA methylated by MET1 independently of HDA6. On the other hand, we found that the target loci in Group A (with loss of DNA methylation in *axe1-5*) were isolated from other DNA methylated regions as no substantial methylation was detected around the target loci (Figure 6C, Group A). We confirmed the absence of DNA methylation around the HDA6 target loci in Group A by bisulfite sequencing analysis of the upstream region of a Group A gene, *At5g41660* (data not shown). Thus, we deduced a requirement for HDA6 involvement in CG methylation by MET1 for HDA6 target loci, in the absence of other flanking DNA methylated regions.

## Discussion

We have identified 157 loci that require HDA6 for epigenetic silencing in *Arabidopsis*. This is the first report to identify derepressed loci in *axe1-5* on a genome-wide scale. Our study revealed several interesting features of HDA6 target loci, mapped large numbers of TE fragments and DNA methylation sites in wild-type *Arabidopsis* plants. The derepressed loci in *axe1-5* overlapped significantly with the derepressed loci in *met1-3*, a CG DNA methyltransferase mutant, rather than the RdDM deficient mutants *rdr2* and *ddc*, suggesting that HDA6 plays an important role in gene silencing, in cooperation with MET1. We also identified 17 direct targets of HDA6 using ChIP-qPCR assays with a HDA6 specific antibody. We found that HDA6 was required for heterochromatic histone modifications and DNA methylation in the target loci. Interestingly, HDA6 deficiency resulted in aberrant enrichment for euchromatic epigenetic marks and DNA hypomethylation at HDA6 targets, along with ectopic expression of these loci. DNA hypomethylation at CG sites in *axe1-5* occurred at some HDA6 target loci, but only where isolated from other MET1 target loci, possibly indicating the requirement of HDA6 for the recruitment of MET1 to specific loci.

We showed that all of the HDA6 direct targets tested in this study colocalized with a constitutive heterochromatin mark, histone H3K9me2 (7 out of 7; Figure 4C), rather than another mark of repressive chromatin, H3K27me3 (Figure 4D, 3 out of 7). We saw no evidence of euchromatic modification H3K4me3 (0 out of 7; Figure 4B). Moreover, our results suggested the deacetylase activity of HDA6 against H3K9ac and H3K27ac, will be important for subsequent histone methylations of H3K9me2 and H3K27me3, as well as H3K14ac, H3K18ac, H3K23ac, H4K5ac, H4K8ac and H4K12ac. These results suggest that H3K9 and H3K27 deacetylation by HDA6 is essential for the establishment of the heterochromatic and repressive marks mediated by H3K9me2 and H3K27me3, and HDA6 deficiency resulted in loss of heterochromatic histone marks and aberrant enrichment for euchromatic marks at HDA6 target loci. It is noteworthy that the enrichments observed on H3K23ac were the highest among the possible acetylation sites, which may indicate the importance of H3K23 deacetylation on heterochromatic gene silencing. The abundance of silenced TE fragments and genes for unknown proteins among the loci derepressed in *axe1-5* (Figure 1C and 1D) supports the role of HDA6 in silencing at heterochromatic regions. In addition, an important role for HDA6 in CHG methylation is indicated by the observation that all of the direct targets of HDA6 tested in this study lost CHG methylation in *axe1-5* (Figure 6B; Figure S6). Several papers report that CHG methylation maintained by CMT3 is dependent on H3K9me2 and that CMT3 is recruited to methylated histones [24]–[26]. Taken together, this indicates that HDA6 deacetylase activity against its target loci is required for establishment of the heterochromatic and repressive marks H3K9me2 and H3K27me3, and CHG methylation by CMT3.

The separation of endogenous HDA6 target loci from endogenous RdDM target loci were investigated in this study. Our results show that endogenous HDA6 target loci were associated with a constitutive heterochromatic mark, H3K9me2 (Figure 4C), but not a euchromatic mark, H3K4me3 (Figure 4B). However, many of the endogenous target loci of RdDM components (such as Pol V and DRD1) were found to be associated with euchromatic histone modification H3K4me3, but not H3K9me2 [45]. Surprisingly, genome-wide identification of the loci derepressed in *axe1-5* revealed that these loci overlap with only a small fraction of the genes upregulated in *rdr2* (Figure 2A). However, considering the recent studies proposing the role of siRNAs in re-establishment of DNA methylation and gene silencing when DNA methylation was lost in the DNA methylation deficient mutants like *met1* and *ddm1* [48]–[50], the siRNAs found on the HDA6 target loci might also have a role in this mechanism, and therefore the double mutants of *hda6* and siRNA deficient mutants might result in larger release of gene silencing. In addition, cell-type specific regulation of siRNAs and TEs silencing especially in the gametes has been proposed recently [51]. In this case, *DDM1* expression is downregulated in the pollen vegetative nucleus, which accompanies the sperm cells. Considering that HDA6 has common features with DDM1 [18], [29], [36]–[38], it would also be interesting to see if HDA6 also have a role in regulating transposon silencing in gametes.

**Figure 7:**
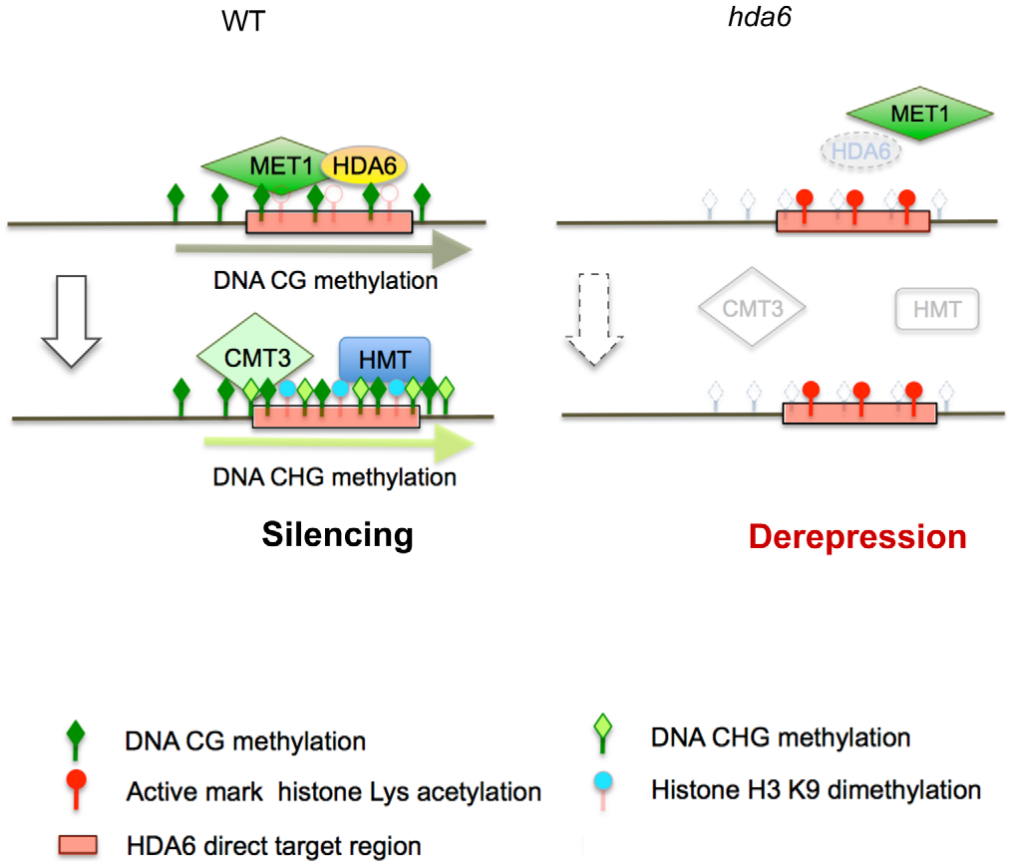
Mechanistic model for epigenetic heterochromatin silencing regulated by HDA6. In our hypothetical model, HDA6 and MET1 cooperate in the initial step of epigenetic silencing. HDA6 directs deacetylation of all lysine residues in H3 and H4 N-tails, except H4K16. The CG DNA methyltransferase MET1 probably requires HDA6 for its recruitment to the HDA6 target loci isolated from other MET1 target loci. Once MET1 is recruited it directs CG DNA methylation. After deacetylation by HDA6 and CG methylation by MET1, H3K9me2 is established by Histone Methyl Transferase(s) (HMT), followed by H3K9me2-dependent CHG methylation by CMT3. In *hda6*, all repressive modifications such as H3K9me2, histone deacetylation, and all DNA methylation in all sequence contexts are lost. This results in transcriptional derepression, indicating that HDA6 may trigger a series of epigenetic modifications at heterochromatin, possibly as a recruiter, or as a component of the silencing machinery in conjunction with MET1.

An important functional connection between HDA6 and MET1 was revealed by observing the significant overlap of the loci upregulated in *axe1-5*, with the loci upregulated in *met1-3* (Figure 2B and 2C). Indeed, we found several HDA6 target loci (AT3TE60310, AT3TE76225, *At3g54730* and *At5g41660*) that require HDA6 for MET1 CG methylation (Figure 6B; Group A). Thus, we propose that HDA6 acts in cooperation with MET1, possibly as a recruiter or as a component of the silencing machinery with MET1 (Figure 7). Actually we observed the loss of HDA6 binding on several HDA6 targets in the *met1-3* mutant (Figure S10). It indicates the requirement for MET1 and/or CG methylation to facilitate HDA6 binding, suggesting the cooperative interplay between HDA6 and MET1. Further support for this hypothesis, is the observation that HDA6 targets isolated from other flanking MET1 targets experienced a loss of CG methylation in the absence of HDA6 (Figure 6B and 6C). It is also supported by several papers evidencing the physical interactions that occur between histone deacetylases and DNA methyltransferases in mammals [52]–[54].

The sustained CG methylation status of the other HDA6 targets in the *axe1-5* mutant was found to correlate with the existence of other flanking MET1 target loci in neighboring regions of the HDA6 targets (Figure 6B and 6C, Group B; Figure S7, S8, S9, S11). Thus it appears likely, therefore, that MET1 could be recruited to the neighboring regions of the HDA6 targets and pass through the HDA6 targets (Figure S11). Another possibility is that MET1-dependent CG methylation is the primary repressive modification to silence those HDA6 targets. There also were many loci that were derepressed only in the *met1-3* mutant (Figure 2C, Figure S12).

It is noteworthy that the intensities of the RT-PCR bands were greater in *met1-3* than in *axe1-5* for the genes with sustained CG methylation (i.e. *At1g67105*, AT3TE63935, *At2g15555*, *At3g44070*, and *G683*; Figure 2B), indicating that sustained CG methylation on the HDA6 target loci can be repressive to some extent. However, there is clear evidence that these genes are transcriptionally derepressed strongly in the absence of HDA6 or MET1, regardless of the CG methylation status. We deduce, therefore, that both HDA6 histone deacetylation and MET1 CG methylation are essential for the silencing of HDA6 target loci. In addition, for most of the HDA6 target loci, CHG and CHH methylation was lost in both *hda6* and *met1* mutants (Figure 6B). A drastic reduction in CHG and CHH methylation was also observed for the loci silenced by HDA6 in the *met1* mutant (Figure 2E). These observations indicate that CHG and CHH methylation on HDA6 targets require the presence of epigenetically silent chromatin associated with both histone deacetylation and CG methylation. Because *HDA6* was identified as *RTS1* (RNA-mediated transcriptional silencing 1) and *MET1* as *RTS2* in RdDM screens [22], [27], we strongly suggest that these two genes are deeply connected to each other, as proposed previously [22], [36]. These insights have an important evolutionary implication; that the histone deacetylase superfamily, one of the most ancient enzymes in eukaryotes [16], may build the foundations for gene silencing and concomitant CG DNA methylation. CG DNA methylation is a conserved modification in higher eukaryotes, quite distinct from the siRNA derived CHG or CHH methylation found only in plants [55], [56].

It is noteworthy that HDA6 has high specificity for target loci within the genome. Moreover, we found that HDA6 binds only to its target loci, not the flanking TE fragments (Figure S9). Relatively low numbers of loci were derepressed in *axe1-5* (157 loci), contrasting with the *met1-3* mutant, where a total of 1215 loci were derepressed. We consistently detected an insignificant difference in the amount of total methylated DNA in *axe1-5*, whereas a severe reduction was detected in the *met1-3* mutant compared with wild-type plants (Figure S13). It was also reported that the total amount of histone H4 tetra-acetylation or H3K4me3 did not change between *axe1-5* and the wild-type plants [28]. From this, we conclude that the target specificity of HDA6 is likely to be precisely controlled, with the effect of the *hda6* mutation manifesting itself only in local areas. Furthermore, HDA6, but not MET1, has the ability to trigger *de novo* chromatin silencing, because only backcrossed *hda6*/wild-type plants were able to restore DNA methylation, low H3K4me3 levels and a silent transcriptional state, comparable with wild-type plants, unlike *met1*/wt [36], [37]. These findings, taken together, indicate that HDA6 is a regulator of locus-directed heterochromatin silencing in cooperation with MET1, where it acts possibly as a recruiter or as a component of the chromatin silencing machinery with MET1, thus establishing the foundations for silent chromatin status for the subsequent heterochromatin mark H3K9me2 and non-CG methylation (Figure 7).

Because MET1 is the primary CG DNA methyltransferase encoded in *Arabidopsis*, regulation of the proper and specific distribution of MET1 CG methylation by employment of HDA6 and/or other possible factors would be an efficient way for the *Arabidopsis* genome to adapt to several developmental and environmental effects. It will be interesting to see if HDA6 and MET1 form a complex as is the case in mammals [52]–[54], or what information is recognized by these factors to trigger the sequential silencing mechanism. In this study, we identified dozens of loci transcriptionally silenced by HDA6 and several loci directly targeted by HDA6. These results will undoubtedly contribute to an understanding of the complex interplay between histone deacetylation and DNA methylation, revealing mechanistic insights into heterochromatin silencing in higher eukaryotes.

## Materials and Methods

### Plants and Growth Conditions

Seeds were surface-sterilized and stratified for 4 days at 4°C in the dark. The seeds were then grown in tissue culture plates on MS agar (0.8%) medium supplemented with 1% sucrose under 16 h light/8 h dark for 15 days at 22°C. All experiments used *axe1-5* [33], *met1-3* [21], *ddc* (*drm1-2 drm2-2 cmt3-11*, [57]), *kyp* (SALK_069326 [58]), *rdr2-1* (SAIL_1277_H08 [42]), *nrpd1a-3* (SALK_128428 [59]) mutants or DR5 [33] and Col-0 wild-type plants. All plants were the Columbia ecotype.

### Whole-Genome Tiling Array Analysis

The GeneChip Arabidopsis tiling array set (1.0F Array and 1.0R Array, Affymetrix) was used. Total RNA was extracted using Isogen reagent (Nippon Gene). Probe synthesis, hybridization, detection, data evaluation with U-test (FDR α = 0.05) and P initial value (P<10^−6^) was conducted essentially as described previously [60]. Three independent biological replicates were performed for each strand array. Detection of intergenic transcribed units was performed as described previously [39], [60] based on the TAIR8 annotation. A threefold increase or decrease in RNA accumulation was taken as additional criteria for defining the loci upregulated or downregulated in the *axe1-5* and *met1-3* mutants. Tiling array data are available at the GEO website under the accession number GSE23950.

### RT-PCR Analysis

Total RNA was extracted using Plant RNA Purification Reagent (Invitrogen) and subjected to cDNA synthesis using the QuantiTect Reverse Transcription Kit (Qiagen), according to the manufacturer's instructions. The PCR conditions were as follows; pre-incubation for 5 min at 94°C, 30 cycles at 94°C for 30 sec, 58°C for 20 sec, 72°C for 40 sec and a final extension at 72°C for 4 min. Primers are listed in Table S8. The amplified DNA was visualized on a 2% agarose gel stained with ethidium bromide.

### Generation of the HDA6 Antibody and Western Blot Analysis

Antibodies against HDA6 were generated as follows; a peptide (DEMDDDNPEPDVNPPSS) corresponding to the C-terminus of HDA6 was synthesized, HPLC purified, conjugated to Bovine Serum Albumin (BSA) and used to immunize two rabbits (Scrum). The antiserum obtained was affinity-purified and used for ELISA and western blot analysis. Total protein extraction was performed on 15-day-old seedlings. Seedlings were ground in liquid nitrogen, suspended in PBS supplemented with 1 mM PMSF, centrifuged, and the supernatant used as a total protein extract. The protein concentration was analyzed using the BioRad Bradford reagent and 50 ug of protein was used for western blot analysis. Western blots prepared by the iBlot Dry Blotting system (Invitrogen) were blocked and incubated with the HDA6 antibody diluted at 1∶500, washed, and incubated with anti-rabbit IgG HRP-conjugated antibodies (GE Healthcare) diluted 1∶5000. The results were visualized using ECL Plus Western Blotting Detection Reagents (GE Healthcare).

### Chromatin Immunoprecipitation

ChIP assays were performed essentially as described previously [61]. The antibodies used in this study were: anti-H3K4me3 and H3K9me2 [62]; anti-H3K9ac (ab4441) and H3K14ac (ab1191) from Abcam; anti-H4 tetra-acetylation (06-866), H3K27me3 (07-449), H3K18ac (07-328), H3K23ac (07-355), H3K27ac (07-360), H4K5ac (07-327), H4K8ac (07-328), and H4K12ac (07-595) from Millipore, and H4K16ac (CB-SC-8662-R) from Santa Cruz. The precipitates were analyzed with quantitative PCR (Power SYBR real time reagent and ABI Prism 7000, Applied Biosystems) and the relative amount of each modification was estimated as described previously [63]. Statistical significance of the wild-type plants compared with *axe1-5* was determined by Kruskal–Wallis test (P<0.05). The primers used are listed in Table S8.

### DNA Methylation Analysis

Genomic DNA was extracted using a Phytopure DNA extraction kit (GE Healthcare) and 5 µg of genomic DNA was linearlized with 20 U *Bam*HI for 3 hours at 37°C. McrBC assays were performed by incubating 30 U of McrBC per 1 µg of *Bam*HI digested genomic DNA at 37°C for 16 hours before PCR amplification as described for RT-PCR with a 1 min extension time. Chop-PCR assays [30] were performed using the methylation sensitive restriction enzymes *Hpa*II, *Msp*I, and *Hae*III (NEB). Linearlized genomic DNA was incubated with the enzymes (30 U/µg) at 37°C for 3 hours and subjected to PCR analysis. The amplified DNA was visualized on a 1.0% agarose gel stained with ethidium bromide.

## Supporting Information

Figure S1 Validation of up- or down-regulation of selected AGI genes and non-AGI TUs in *axe1-5* by RT-PCR. Several loci that were differentially expressed in *axe1-5* in the tiling array analysis were randomly selected and their up- or down-regulation confirmed by RT-PCR. 24 AGI genes and 4 non-AGI TUs were used. *ACT2* and *At5g55670*, which showed no transcriptional change in the tiling array analysis, were used as controls. Primers are listed in Table S8.
(TIF)

Figure S2 The numbers of siRNA sequences in wild-type plants that correspond to the loci upregulated in *axe1-5*. siRNA sequences of inflorescences of the wild-type, *rdr2*, and *dcl3* plants were retrieved from the ASRP database. (A) The numbers of siRNA sequences in wild-type plants that correspond to the loci upregulated in *axe1-5*. (B) The numbers of 24-nt siRNAs and 21-nt siRNAs sequences in wild-type, *rdr2* and *dcl3* plants corresponding to the loci upregulated in *axe1-5*. (Black, Col-0; blue, *rdr2*; yellow, *dcl3*).
(TIF)

Figure S3 Search for the binding position of HDA6 in target genes. Direct binding of HDA6 within the promoter, 5′ and 3′ transcribed regions of three representative derepressed genes, AT3TE60310, AT3TE76225, and *At5g41660* were examined using ChIP-qPCR assays with the HDA6 antibody. Equal amount of input DNA and the immunoprecipitates were analyzed and normalized against input DNA and *ACT2*. The relative enrichment of HDA6-binding in the wild-type against that in *axe1-5* is shown as the mean of the results of repeated experiments with three independent immunoprecipitated DNA preparations (orange, WT; green, *axe1-5*). Error bars indicate the standard deviation.
(TIF)

Figure S4 Histone modification status as determined by ChIP-PCR with specific antibodies for H4 tetra-acetylation, H3K4me3, H3K9me2 and H3K27me3. Three representative HDA6 direct targets (AT3TE60310, AT3TE76225, and *At5g41660*) were examined. *ACT2* served as a control. Equal amounts of the input and the immunoprecipitated DNA were subjected to 30 cycles of PCR amplification using the same primers as in Figure 3C. PCR amplicons were analyzed by 6% polyacrylamide gel electrophoresis.
(TIF)

Figure S5 Enrichments of histone acetylation in *axe1-5* were examined by ChIP-PCR with specific antibodies for all the possible substrates of HDA6 deacetylation at H3 and H4 N-tails. The acetylation levels of AT3TE60310, AT3TE76225 and *At5g41660* were analyzed using *ACT2* as a control. Equal amount of the input and the immunoprecipitated DNA were subjected to 30 cycles of PCR using the same primers used in Figure 3C. The PCR products obtained were analyzed by 6% polyacrylamide gel electrophoresis.
(TIF)

Figure S6 DNA methylation status of HDA6 target loci in wild-type, *axe1-5* and *met1-3* determined by bisulfite sequencing. Representative HDA6 target loci from each group (*At5g41660* and AT3TE76225 from Group A; AT3TE63935 from Group B) were analyzed for their DNA methylation status by bisulfite sequencing analysis. (A) The percentage total cytosine methylation is shown as the mean and standard deviation of 10 independent sequencing reads (red, CG methylation; blue, CHG methylation; green, CHH methylation). (B) The percentage of methylated cytosine at each site was analyzed using publicly available software: Kismeth (http://katahdin.mssm.edu/kismeth). The primers used are listed in Table S8. Bisulfite treatment was performed using BisulFast DNA modification Kit for Methylated DNA Detection (TOYOBO). The modified DNA was amplified as follows by PCR: pre-incubation step of 1 min at 94°C, 40 cycles at 94°C for 20 sec, 50 to 54°C for 20 sec, 72C for 1 min and a final extension for 4 min. at 72°C, using Ex-Taq polymerase (Takara Bio). The amplified DNA was cloned into pCR4 using a TOPO TA cloning kit (Life Technologies), transformed into *E. coli* DH5α cells and plasmid DNA purified from single colonies for sequencing.
(TIF)

Figure S7 DNA methylation status of HDA6 target loci and their surrounding regions. The DNA methylation status of the HDA6 direct targets and their surrounding regions were investigated by reference to GBrowse (http://gbrowse.arabidopsis.org), which shows the DNA methylation datasets of HMBD [9]. Upstream and downstream regions of representative HDA6 target loci from each group are shown. The yellow arrow indicates the HDA6 target loci.
(TIF)

Figure S8 DNA methylation of the TE fragments located adjacent to the HDA6 target loci in Group B loci is dependent on MET1, but not HDA6. The DNA methylation status of the TE fragments located adjacent to the HDA6 target loci in Group B was determined using McrBC assays. The TE fragments located adjacent to the HDA6 target loci in Group B are shown in the schematic diagram (orange box). The inside of the TE fragments were investigated (green arrow). McrBC-digested genomic DNA was PCR amplified using the primer sets listed in Table S8.
(TIF)

Figure S9 ChIP-qPCR assay showing that some TE fragments located adjacent to the HDA6 targets are not directly targeted by HDA6. HDA6 binding to the TE fragments located adjacent to the HDA6 target loci in Group B (AT3TE63935, AT5TE43385, *At1g67105*, and *At3g44070*) was examined using ChIP-qPCR. Relative enrichments of HDA6-binding in wild-type plants versus *axe1-5* are shown as the mean plus standard deviation of three independent immunoprecipitates.
(TIF)

Figure S10 ChIP-qPCR assay for the HDA6 binding at some HDA6 direct targets in the *met1-3* mutant. HDA6 binding to the HDA6 direct targets (AT3TE60310, *At3g54730*, *G683* and *G1136*) was examined using ChIP-qPCR. Relative enrichments of HDA6-binding in wild-type plants versus *met1-3* are shown.
(TIF)

Figure S11 Model for the epigenetic mechanism of heterochromatin silencing regulated by HDA6 on HDA6 target loci surrounded by other MET1 target loci. HDA6 is required for the maintenance of epigenetic chromatin modifications such as H3K9me2, CHG and CHH DNA methylation, but not required for the recruitment of MET1, when MET1 target loci are located adjacent to HDA6 target loci. HDA6 directs deacetylation of all lysine residues in H3 and H4 N-tails, except H4K16. H3K9me2, CHG and CHH DNA methylation were all dependent on HDA6. However, once MET1 is recruited near HDA6 target loci, MET1 directs CG DNA methylation on or around the HDA6 target loci even in the absence of HDA6. Thus, in *hda6*, repressive modifications such as H3K9me2, histone deacetylation, and non-CG methylation are lost; only CG methylation was retained on the HDA6 target loci. Even though CG methylation may or may not be sustained, transcriptional derepression occurred in *hda6* and *met1*, indicating the requirement for both histone deacetylation by HDA6 and CG methylation by MET1 for establishment of the silent heterochromatin status.
(TIF)

Figure S12 Validation of genes upregulated only in *met1*-*3* by RT-PCR. Several loci that were upregulated in *met1*-*3* but not in *axe1-5* in the tiling array analysis were selected and their upregulation was confirmed by RT-PCR. 4 AGI genes were used. Primers are listed in Table S8.
(TIF)

Figure S13 Dot blot assay to determine total DNA methylation in wild-type plants and *axe1*-*5*, *met1-3* and *ddc* mutants. Equal amounts of genomic DNA (400 ng) from each genotype was blotted onto Nylon membrane (Hybond N+; GE Healthcare) and incubated with an antibody against 5- Methylcytidine (BI-MECY-0100; EUROGENTEC), followed by a secondary antibody conjugated to HRP. The luminescence of HRP was detected with an ECL detection kit and Hyperfilm ECL (GE Healthcare).
(TIF)

Table S1 AGI annotated genes upregulated in *axe1-5*. The AGI genes that were transcriptionally upregulated in *axe1-5* compared with wild-type plants (>3 fold, p-initial<10^−6^, FDR α = 0.05) are listed.
(XLS)

Table S2 Non-AGI TUs upregulated in *axe1-5*. Among the Non-AGI TUs identified using the ARTADE program, the non-AGI TUs that were transcriptionally upregulated in *axe1-5* compared with wild-type plants (>3 fold, p-initial<10^−6^, FDR α = 0.05) are listed.
(XLS)

Table S3 AGI annotated genes downregulated in *axe1-5*. The AGI genes that were transcriptionally downregulated in *axe1*-5 compared with wild-type plants (<1/3 fold, p-initial<10^−6^, FDR α = 0.05) are listed.
(XLS)

Table S4 AGI annotated genes upregulated in *met1-3*. The AGI genes that were transcriptionally upregulated in *met1-3* compared with wild-type plants (>3 fold, p-initial<10^−6^, FDR α = 0.05) are listed.
(XLS)

Table S5 Non-AGI TUs upregulated in *met1-3*. Among the Non-AGI TUs identified using the ARTADE program, the non-AGI TUs that were transcriptionally upregulated in *met1-3* compared with wild-type plants (>3 fold, p-initial<10^−6^, FDR α = 0.05) are listed.
(XLS)

Table S6 AGI annotated genes downregulated in *met1-3*. The AGI genes that were transcriptionally downregulated in *met1-3* compared with wild-type plants (<1/3 fold, p-initial<10^−6^, FDR α = 0.05) are listed.
(XLS)

Table S7 Non-AGI TUs downregulated in *met1-3*. Among the Non-AGI TUs identified using the ARTADE program, the non-AGI TUs that were transcriptionally downregulated in *met1-3* compared with wild-type plants (<1/3 fold, p-initial<10^−6^, FDR α = 0.05) are listed.
(XLS)

Table S8 List of primers. The primers used in this study are listed.
(XLS)
