# Supplementary figures and images for: Arabidopsis HDA6 Regulates Locus-Directed Heterochromatin Silencing in Cooperation with MET1
Source: PLoS Genet. 2011 Apr 28;7(4):e1002055. doi: 10.1371/journal.pgen.1002055 (PMC3084210; doi:10.1371/journal.pgen.1002055)

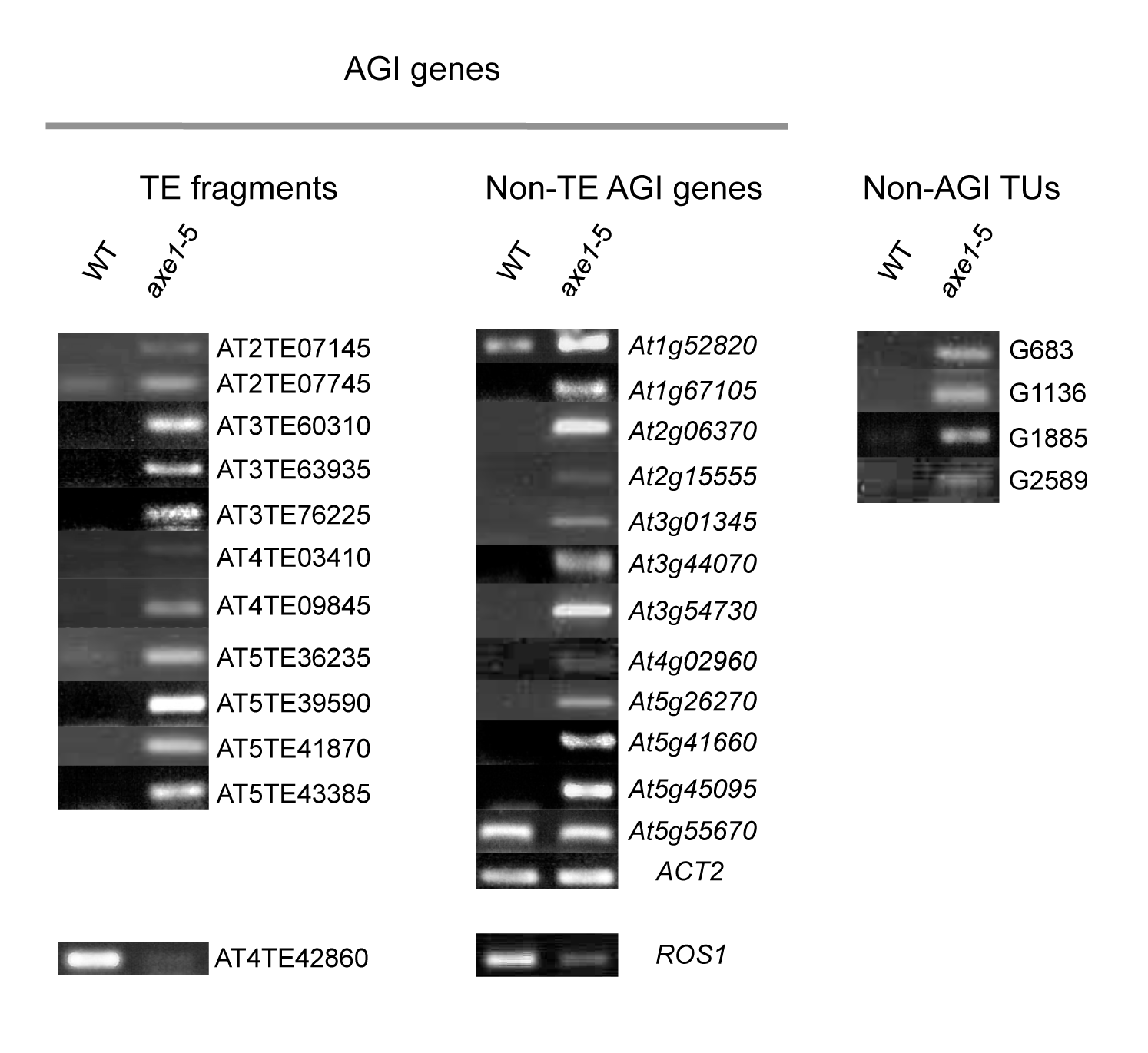

Supplement: Figure S1 — Validation of up- or down-regulation of selected AGI genes and non-AGI TUs in axe1-5 by RT-PCR. Several loci that were differentially expressed in axe1-5 in the tiling array analysis were randomly selected and their up- or down-regulation confirmed by RT-PCR. 24 AGI genes and 4 non-AGI TUs were used. ACT2 and At5g55670, which showed no transcriptional change in the tiling array analysis, were used as controls. Primers are listed in Table S8. (TIF) [file pgen.1002055.s001.tif]

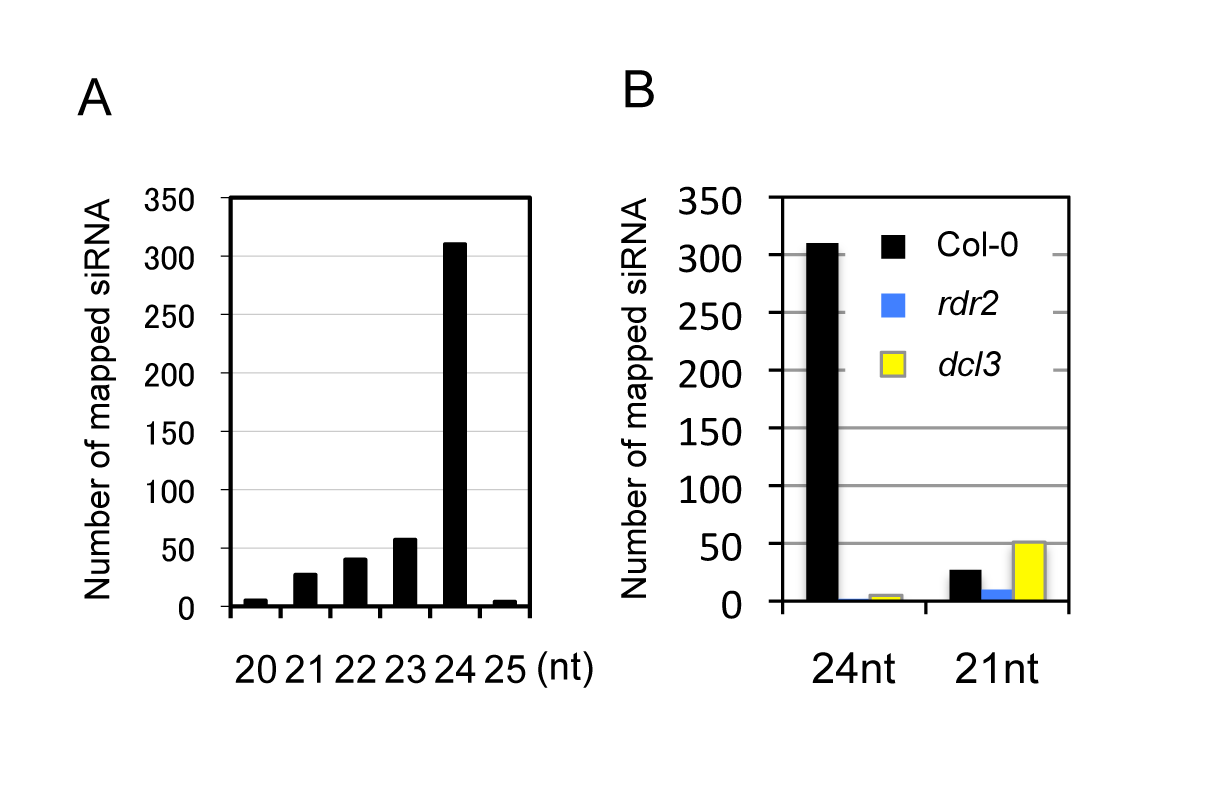

Supplement: Figure S2 — The numbers of siRNA sequences in wild-type plants that correspond to the loci upregulated in axe1-5. siRNA sequences of inflorescences of the wild-type, rdr2, and dcl3 plants were retrieved from the ASRP database. (A) The numbers of siRNA sequences in wild-type plants that correspond to the loci upregulated in axe1-5. (B) The numbers of 24-nt siRNAs and 21-nt siRNAs sequences in wild-type, rdr2 and dcl3 plants corresponding to the loci upregulated in axe1-5. (Black, Col-0; blue, rdr2; yellow, dcl3). (TIF) [file pgen.1002055.s002.tif]

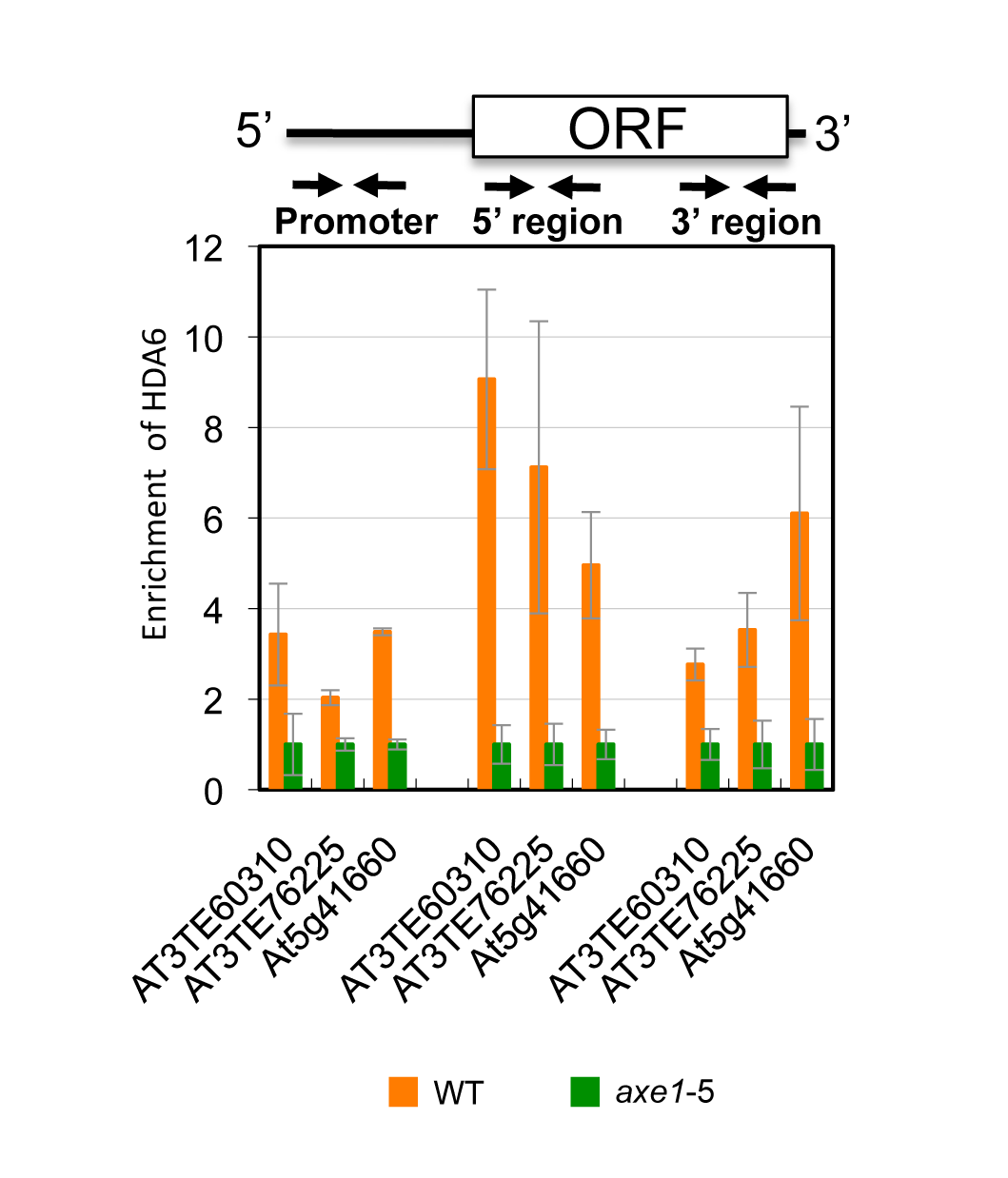

Supplement: Figure S3 — Search for the binding position of HDA6 in target genes. Direct binding of HDA6 within the promoter, 5′ and 3′ transcribed regions of three representative derepressed genes, AT3TE60310, AT3TE76225, and At5g41660 were examined using ChIP-qPCR assays with the HDA6 antibody. Equal amount of input DNA and the immunoprecipitates were analyzed and normalized against input DNA and ACT2. The relative enrichment of HDA6-binding in the wild-type against that in axe1-5 is shown as the mean of the results of repeated experiments with three independent immunoprecipitated DNA preparations (orange, WT; green, axe1-5). Error bars indicate the standard deviation. (TIF) [file pgen.1002055.s003.tif]

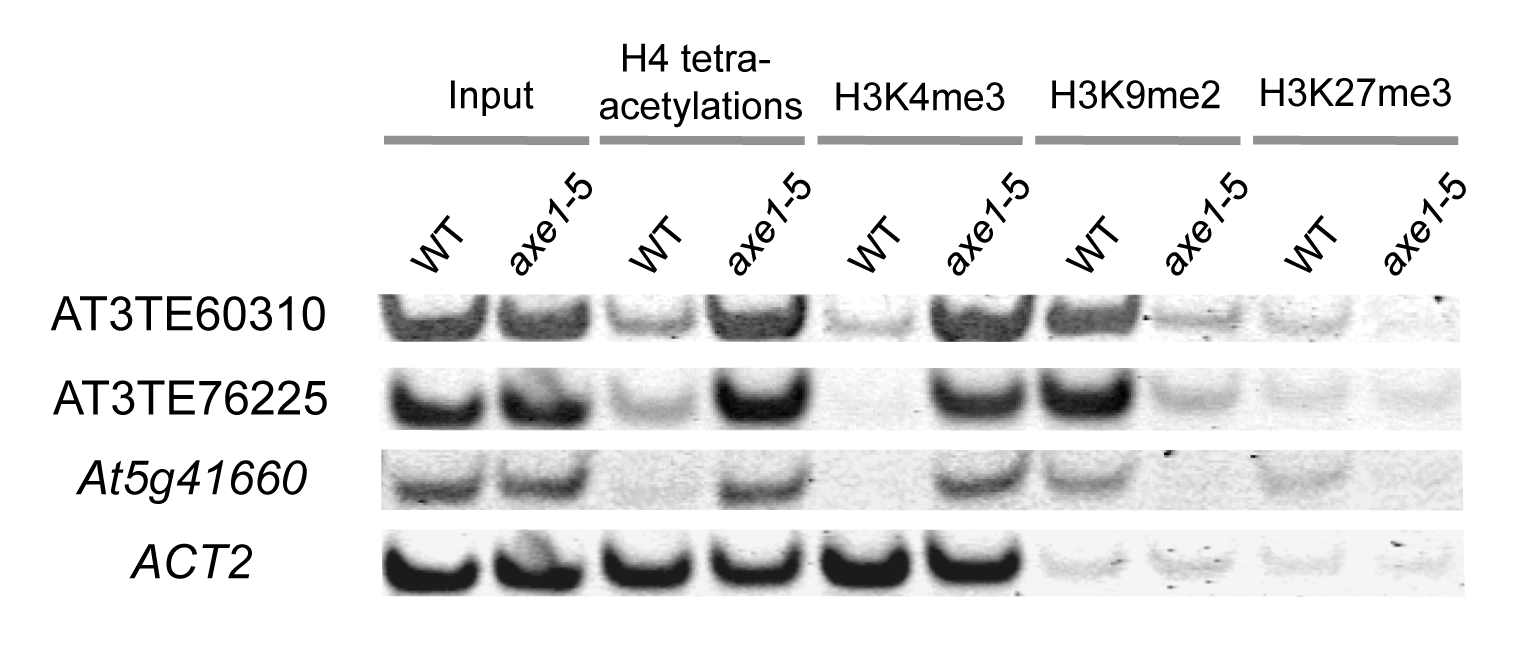

Supplement: Figure S4 — Histone modification status as determined by ChIP-PCR with specific antibodies for H4 tetra-acetylation, H3K4me3, H3K9me2 and H3K27me3. Three representative HDA6 direct targets (AT3TE60310, AT3TE76225, and At5g41660) were examined. ACT2 served as a control. Equal amounts of the input and the immunoprecipitated DNA were subjected to 30 cycles of PCR amplification using the same primers as in Figure 3C. PCR amplicons were analyzed by 6% polyacrylamide gel electrophoresis. (TIF) [file pgen.1002055.s004.tif]

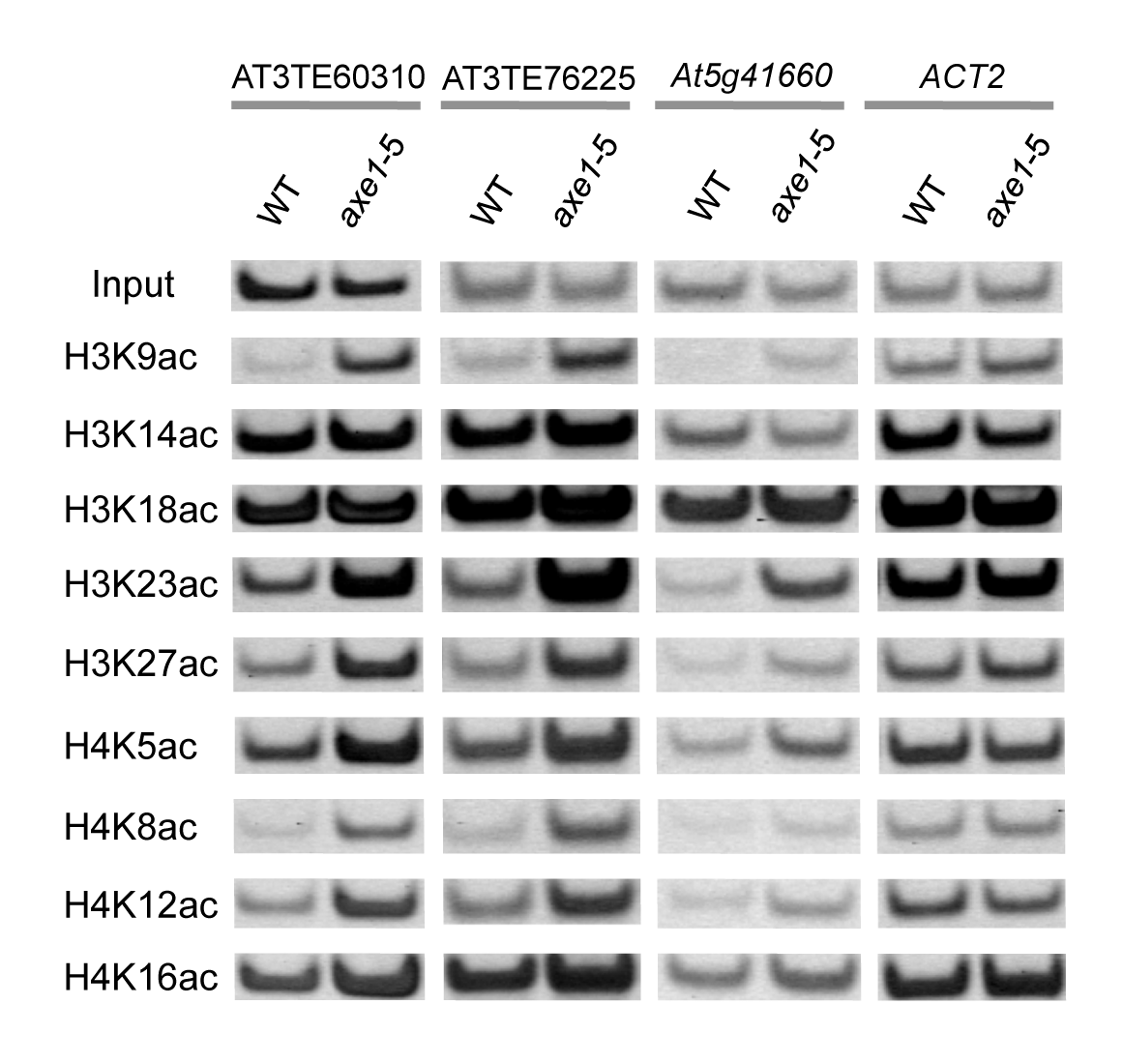

Supplement: Figure S5 — Enrichments of histone acetylation in axe1-5 were examined by ChIP-PCR with specific antibodies for all the possible substrates of HDA6 deacetylation at H3 and H4 N-tails. The acetylation levels of AT3TE60310, AT3TE76225 and At5g41660 were analyzed using ACT2 as a control. Equal amount of the input and the immunoprecipitated DNA were subjected to 30 cycles of PCR using the same primers used in Figure 3C. The PCR products obtained were analyzed by 6% polyacrylamide gel electrophoresis. (TIF) [file pgen.1002055.s005.tif]

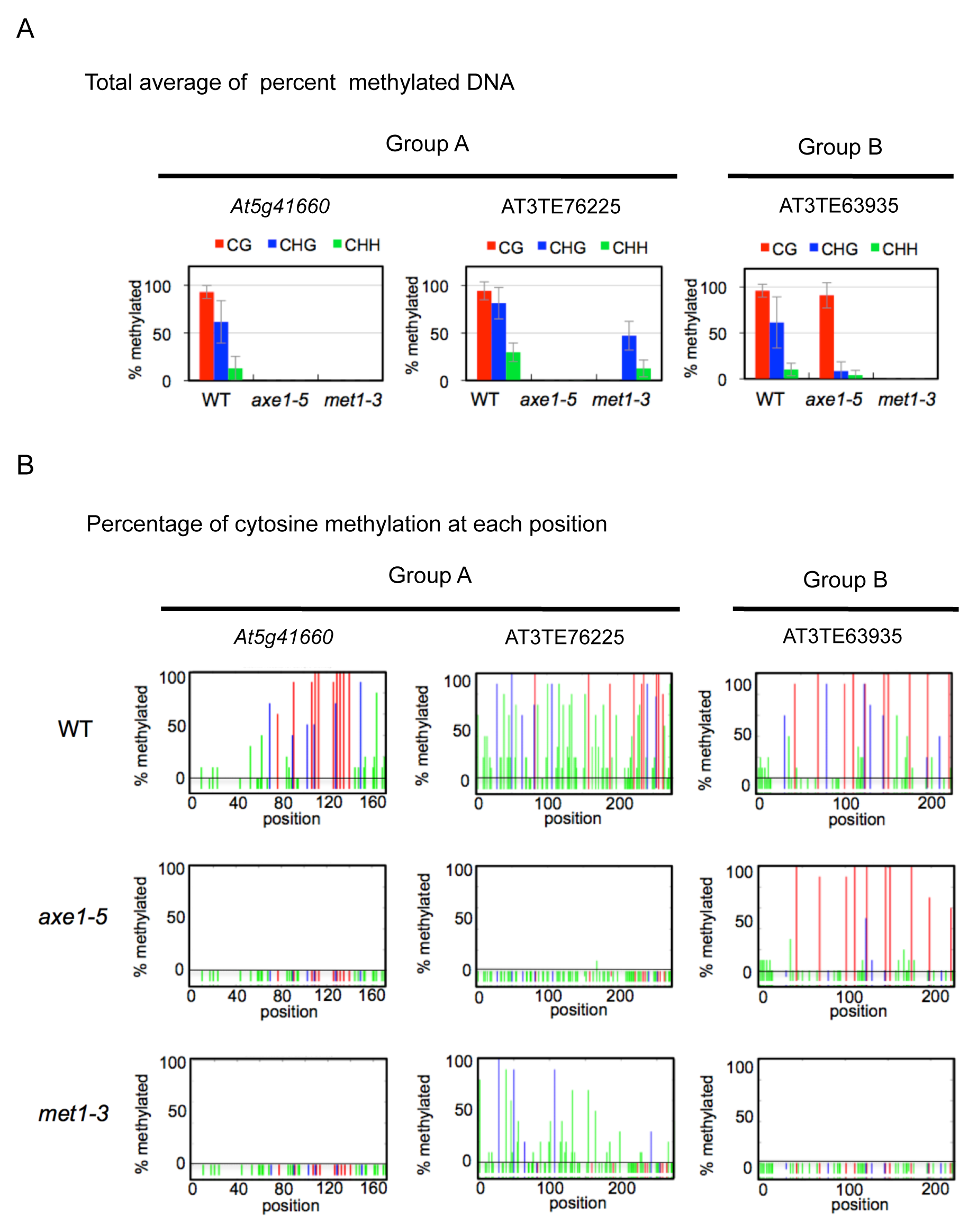

Supplement: Figure S6 — DNA methylation status of HDA6 target loci in wild-type, axe1-5 and met1-3 determined by bisulfite sequencing. Representative HDA6 target loci from each group (At5g41660 and AT3TE76225 from Group A; AT3TE63935 from Group B) were analyzed for their DNA methylation status by bisulfite sequencing analysis. (A) The percentage total cytosine methylation is shown as the mean and standard deviation of 10 independent sequencing reads (red, CG methylation; blue, CHG methylation; green, CHH methylation). (B) The percentage of methylated cytosine at each site was analyzed using publicly available software: Kismeth (http://katahdin.mssm.edu/kismeth). The primers used are listed in Table S8. Bisulfite treatment was performed using BisulFast DNA modification Kit for Methylated DNA Detection (TOYOBO). The modified DNA was amplified as follows by PCR: pre-incubation step of 1 min at 94°C, 40 cycles at 94°C for 20 sec, 50 to 54°C for 20 sec, 72C for 1 min and a final extension for 4 min. at 72°C, using Ex-Taq polymerase (Takara Bio). The amplified DNA was cloned into pCR4 using a TOPO TA cloning kit (Life Technologies), transformed into E. coli DH5α cells and plasmid DNA purified from single colonies for sequencing. (TIF) [file pgen.1002055.s006.tif]

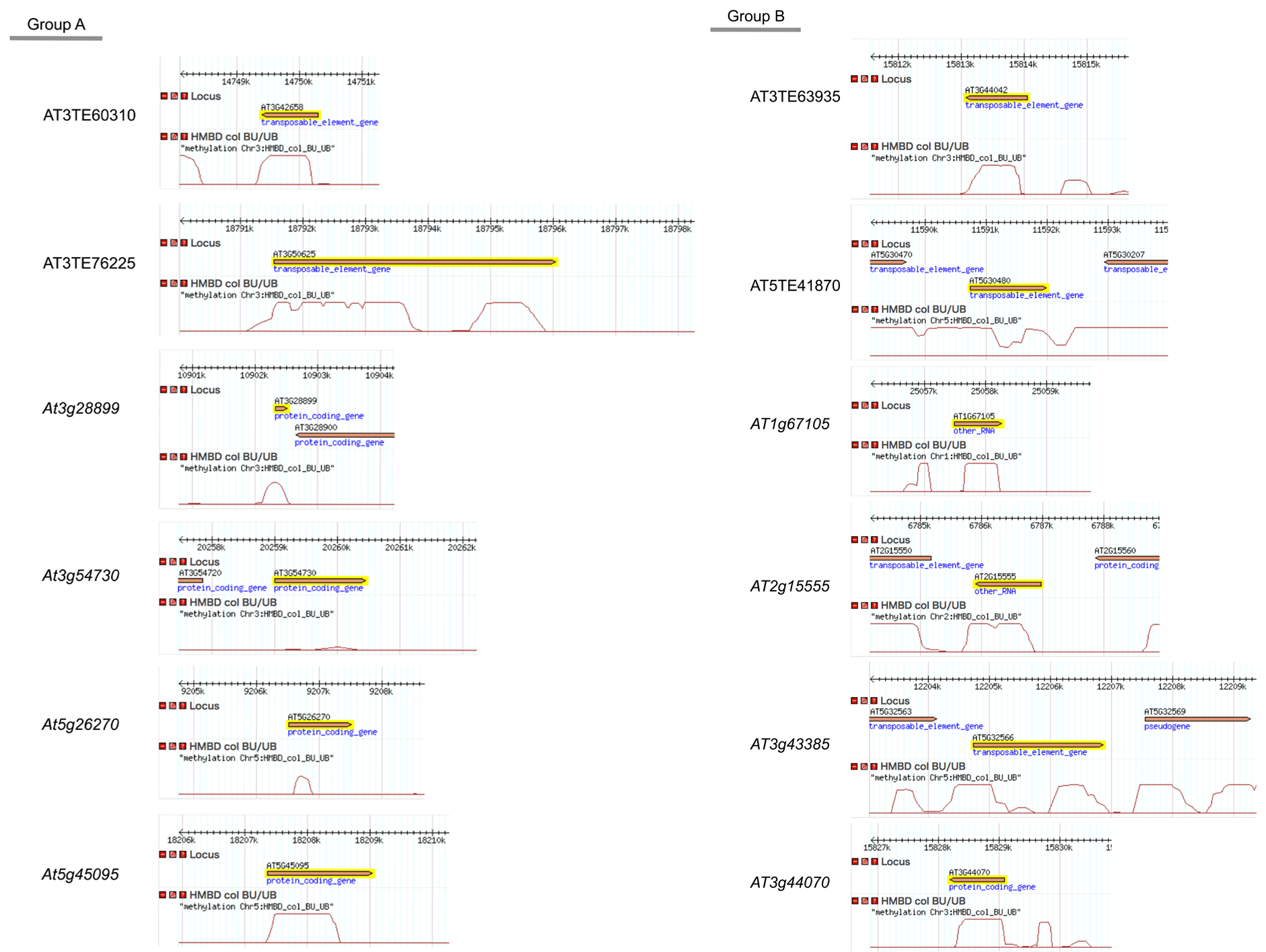

Supplement: Figure S7 — DNA methylation status of HDA6 target loci and their surrounding regions. The DNA methylation status of the HDA6 direct targets and their surrounding regions were investigated by reference to GBrowse (http://gbrowse.arabidopsis.org), which shows the DNA methylation datasets of HMBD [9]. Upstream and downstream regions of representative HDA6 target loci from each group are shown. The yellow arrow indicates the HDA6 target loci. (TIF) [file pgen.1002055.s007.tif]

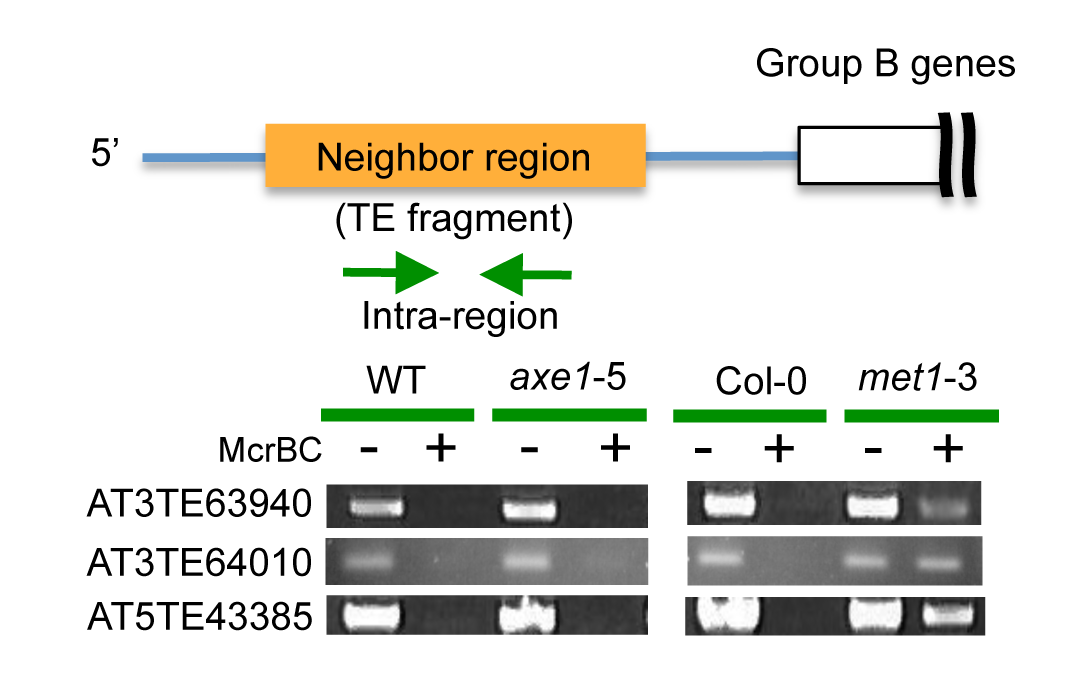

Supplement: Figure S8 — DNA methylation of the TE fragments located adjacent to the HDA6 target loci in Group B loci is dependent on MET1, but not HDA6. The DNA methylation status of the TE fragments located adjacent to the HDA6 target loci in Group B was determined using McrBC assays. The TE fragments located adjacent to the HDA6 target loci in Group B are shown in the schematic diagram (orange box). The inside of the TE fragments were investigated (green arrow). McrBC-digested genomic DNA was PCR amplified using the primer sets listed in Table S8. (TIF) [file pgen.1002055.s008.tif]

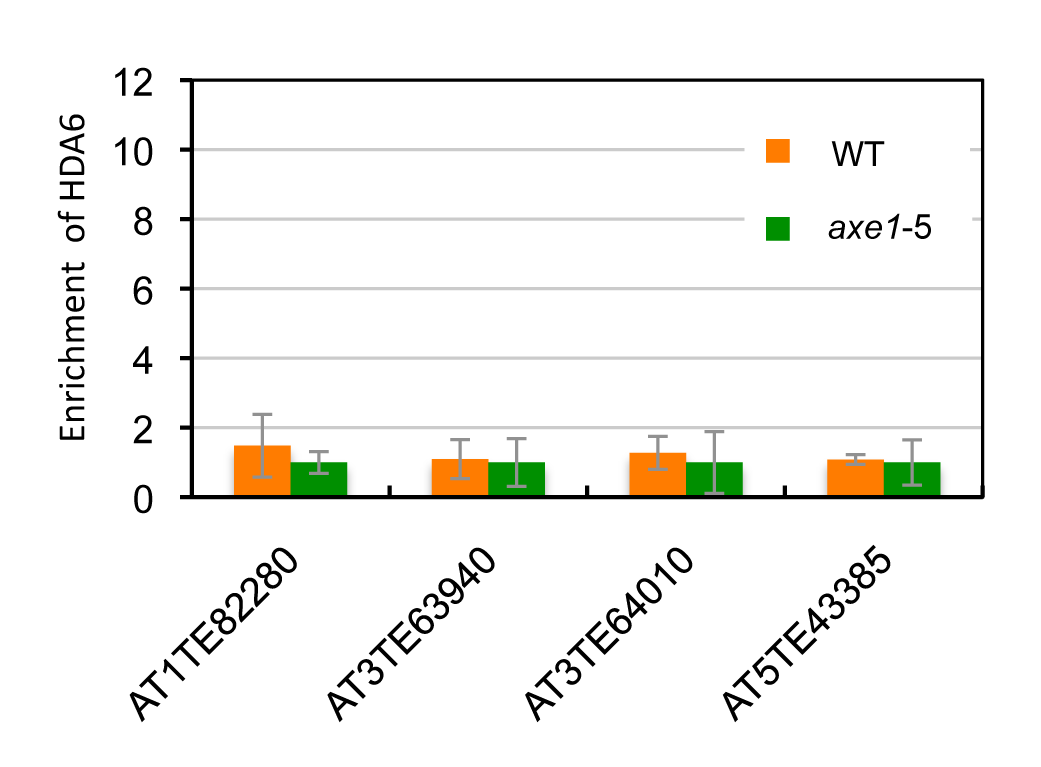

Supplement: Figure S9 — ChIP-qPCR assay showing that some TE fragments located adjacent to the HDA6 targets are not directly targeted by HDA6. HDA6 binding to the TE fragments located adjacent to the HDA6 target loci in Group B (AT3TE63935, AT5TE43385, At1g67105, and At3g44070) was examined using ChIP-qPCR. Relative enrichments of HDA6-binding in wild-type plants versus axe1-5 are shown as the mean plus standard deviation of three independent immunoprecipitates. (TIF) [file pgen.1002055.s009.tif]

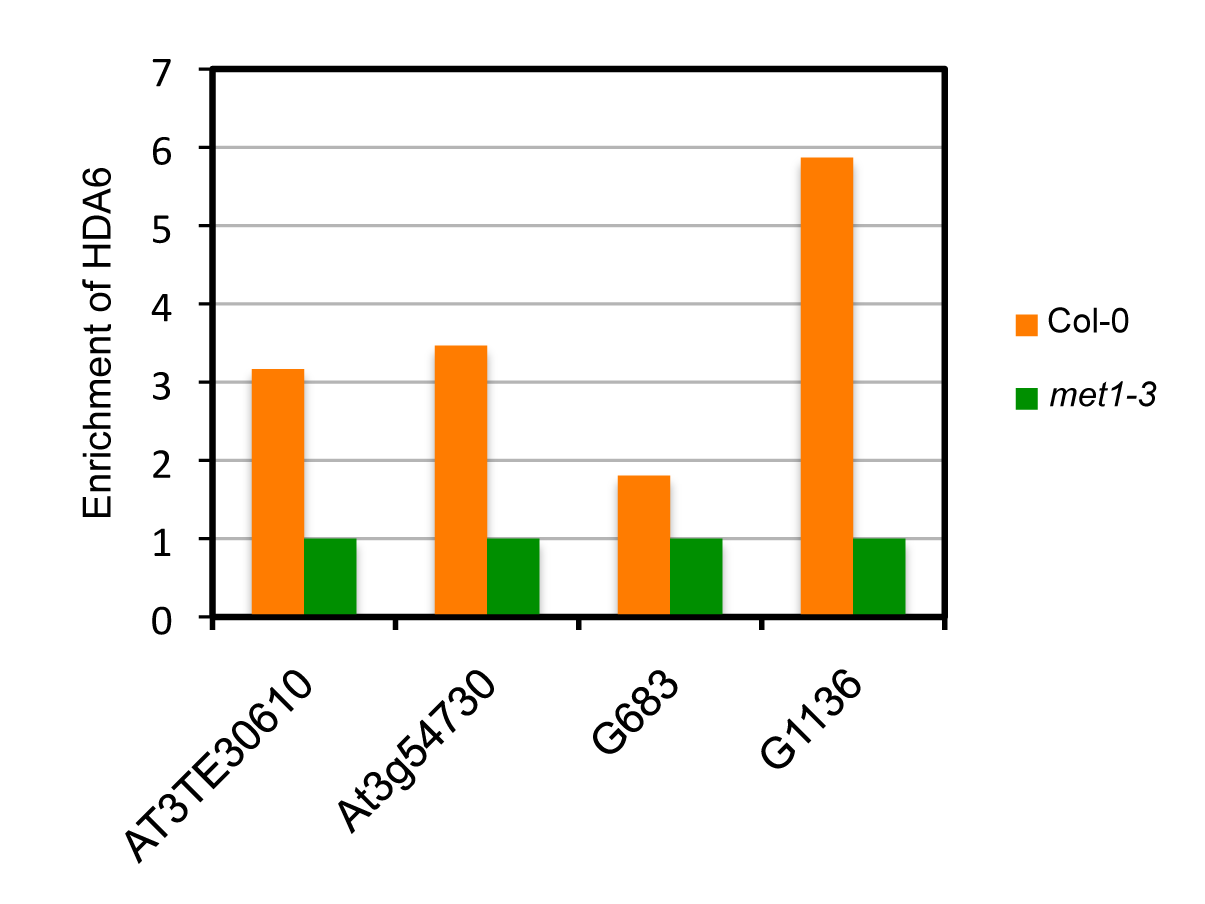

Supplement: Figure S10 — ChIP-qPCR assay for the HDA6 binding at some HDA6 direct targets in the met1-3 mutant. HDA6 binding to the HDA6 direct targets (AT3TE60310, At3g54730, G683 and G1136) was examined using ChIP-qPCR. Relative enrichments of HDA6-binding in wild-type plants versus met1-3 are shown. (TIF) [file pgen.1002055.s010.tif]

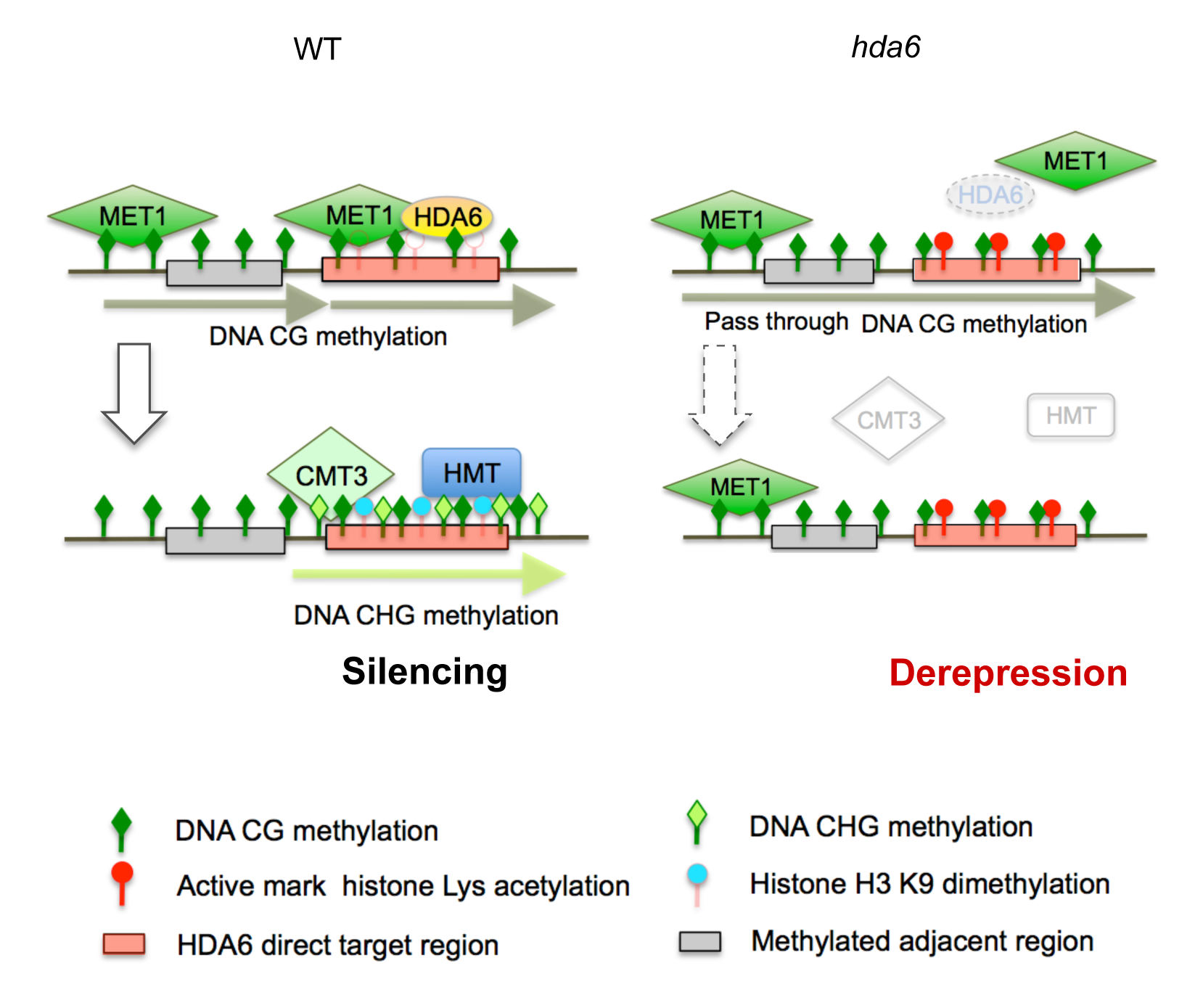

Supplement: Figure S11 — Model for the epigenetic mechanism of heterochromatin silencing regulated by HDA6 on HDA6 target loci surrounded by other MET1 target loci. HDA6 is required for the maintenance of epigenetic chromatin modifications such as H3K9me2, CHG and CHH DNA methylation, but not required for the recruitment of MET1, when MET1 target loci are located adjacent to HDA6 target loci. HDA6 directs deacetylation of all lysine residues in H3 and H4 N-tails, except H4K16. H3K9me2, CHG and CHH DNA methylation were all dependent on HDA6. However, once MET1 is recruited near HDA6 target loci, MET1 directs CG DNA methylation on or around the HDA6 target loci even in the absence of HDA6. Thus, in hda6, repressive modifications such as H3K9me2, histone deacetylation, and non-CG methylation are lost; only CG methylation was retained on the HDA6 target loci. Even though CG methylation may or may not be sustained, transcriptional derepression occurred in hda6 and met1, indicating the requirement for both histone deacetylation by HDA6 and CG methylation by MET1 for establishment of the silent heterochromatin status. (TIF) [file pgen.1002055.s011.tif]

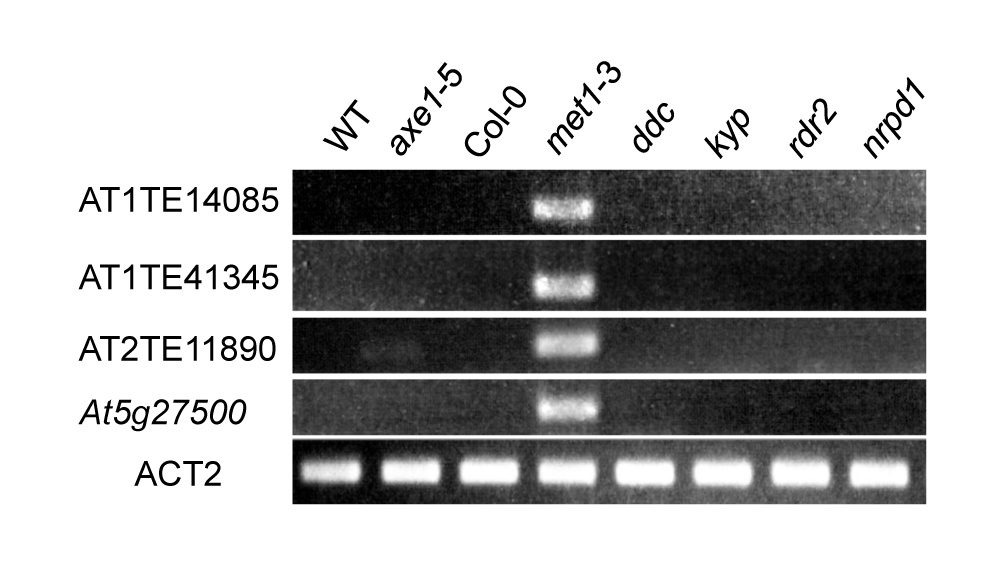

Supplement: Figure S12 — Validation of genes upregulated only in met1-3 by RT-PCR. Several loci that were upregulated in met1-3 but not in axe1-5 in the tiling array analysis were selected and their upregulation was confirmed by RT-PCR. 4 AGI genes were used. Primers are listed in Table S8. (TIF) [file pgen.1002055.s012.tif]

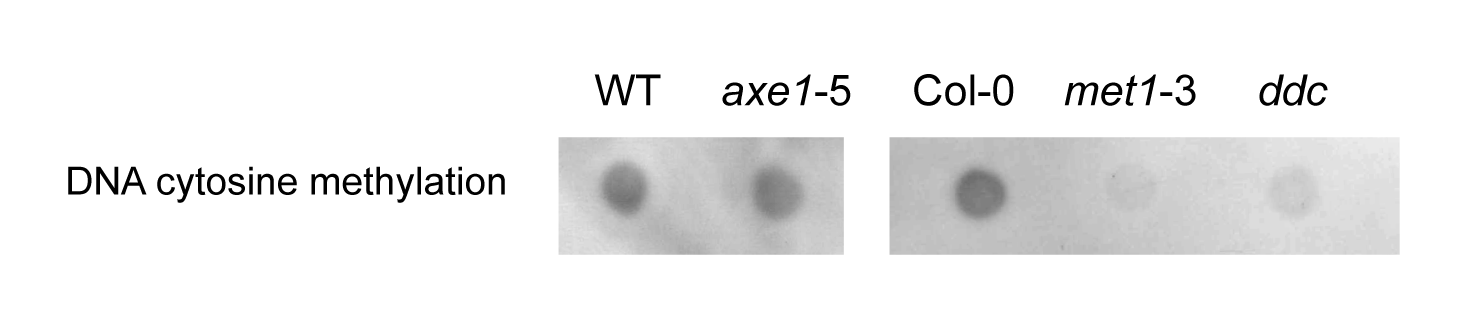

Supplement: Figure S13 — Dot blot assay to determine total DNA methylation in wild-type plants and axe1-5, met1-3 and ddc mutants. Equal amounts of genomic DNA (400 ng) from each genotype was blotted onto Nylon membrane (Hybond N+; GE Healthcare) and incubated with an antibody against 5- Methylcytidine (BI-MECY-0100; EUROGENTEC), followed by a secondary antibody conjugated to HRP. The luminescence of HRP was detected with an ECL detection kit and Hyperfilm ECL (GE Healthcare). (TIF) [file pgen.1002055.s013.tif]
